# RuKY Catalyst‐Packed Permeation Membrane for Quantitative Ammonia and d3‐Ammonia Dehydrogenation to Ultrapure Hydrogen

**DOI:** 10.1002/open.202500480

**Published:** 2026-01-26

**Authors:** Christopher J. Koch, Jennifer Naglic, John T. Kelly, Logan Kearney, José D. Arregui‐Mena, Jochen Lauterbach, Lucas M. Angelette, Tyler Guin

**Affiliations:** ^1^ Savannah River National Laboratory Hydrogen Isotope Processing Science Aiken South Carolina USA; ^2^ Chemical Sciences Division Oak Ridge National Laboratory Oak Ridge Tennessee USA; ^3^ Materials Science and Technology Division Oak Ridge National Laboratory Oak Ridge Tennessee USA; ^4^ Department of Chemical Engineering University of South Carolina Columbia South Carolina USA

**Keywords:** ammonia decomposition, catalysis, fusion, hydrogen carrier, membrane reactor, ruthenium

## Abstract

Ammonia is a promising carbon‐free hydrogen carrier, but incomplete ammonia dehydrogenation (cracking) generates atmospheric emissions of NO_*x*_, a potent greenhouse gas. Additionally, incomplete cracking of ammonia leads to regulatory challenges in nuclear and fusion power, where tritiated ammonia (NT_3_) emissions are strictly controlled. Therefore, we report the use of low‐temperature ammonia dehydrogenation catalysts (3%Ru/1%Y/12%K/Al_2_O_3_) in a palladium alloy H_2_ permeation membrane for quantitative conversion of ammonia into hydrogen and nitrogen at industry‐relevant conditions. This catalytic membrane reactor system achieved an astonishing effluent concentration of <1 ppm at 450°C under a 100% NH_3_ stream, which is far beyond the 99.6% conversion target required for the adoption of ammonia as a vehicle fuel. The low‐temperature ammonia dehydrogenation catalyst was tested in a packed bed reactor with NH_3_ and ND_3_ to both elucidate the reaction mechanism and to quantify the kinetic isotope effect of the membrane reactor. The rate‐limiting step at temperatures relevant to the palladium membrane are isotope independent, indicating that the isotopologue content will not modify the desired reaction kinetics. By reducing emissions to below‐trace levels with no additional separation, this work provides a path to greatly simplified and miniaturized ammonia cracking processes.

## Graphical abstract

The removal of ammonia in gas effluent streams is an important, albeit energy intensive, step in a variety of industrial applications. However, a palladium‐silver permeation membrane reactor greatly increases the performance of the system, allowing for near quantitative conversion of ammonia into nitrogen and hydrogen.

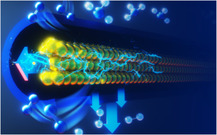

## 1 Introduction

The decomposition of ammonia into hydrogen and nitrogen (“ammonia cracking”) is important for a multitude of utilities, including hydrogen fuel, bioconversion, and now, fusion energy. Ammonia cracking is equilibrium limited, preventing complete conversion and necessitating the venting of trace ammonia or, alternately, expensive separation processes. This difficulty hinders the adoption of ammonia as a hydrogen carrier for fuel, due to venting limits and human proximity to fuel cells. It has been shown that ammonia as a hydrogen carrier would need to have a conversion greater than 99.6% to have lower emissions than greenhouse gases due to the generation of NO_*x*_, which is a hindrance in its overall application as a fuel source [1]. Another critical area for complete conversion of ammonia decomposition is in fusion reactors [2, 3]. Fusion reactors produce tritiated ammonia, NT_3_, from the interaction of tritium with nitrogen at ambient conditions [4, 5, 6]. The formation of NT_3_ leads to problems in the maintenance of such reactors and the loss of valuable tritium [7]. Additionally, tritium and tritiated ammonia cannot be vented into the atmosphere, which poses extreme problems for the adoption of fusion power. Achieving complete conversion of ammonia to nitrogen and hydrogen isotopes is crucial for these industries.

The equilibrium of ammonia cracking can be shifted to complete conversion by removing hydrogen during reaction. This in situ removal can be achieved via the use of a permeation membrane reactor (PMR), in which the walls of the reactor selectively permeate hydrogen. The most common membrane material is a palladium alloy, usually palladium‐silver, which exclusively permeates hydrogen isotopes as a dense membrane [8]. Palladium alloys have relatively low operating temperatures (275°C–500°C), due to propensity to hydride formation and mechanical failure [9, 10, 11, 12, 13]. These PMR systems have been explored in the context of water–gas shift reactions due to the temperature of the reaction being compatible with permeation membranes [14, 15, 16]. Prior efforts in utilizing PMRs for ammonia cracking were limited by the poor performance of the catalyst at the PMR‐relevant temperatures [17, 18, 19, 20, 21]. However, the utilization of permeation membranes for ammonia cracking has been reported [22, 23]. For example, a PMR packed with a traditional Ru/Al_2_O_3_ catalyst utilized only achieves a modern conversion of 98.2% at 1 bar of pressure and 400°C [24, 25]. Even more specialty catalysts, like Ru/La_2_Ce_2_O_7_, only achieve modest conversions (85% under 5 bar of pressure at 400°C) [26]. Importantly, these systems have high pressures of NH_3_ to drive the permeation of hydrogen out of the reactor [25, 27]. However, these types of permeation reactors have trace amounts of N_2_ gas in the permeate that limit its viability in applications, like fusion reactors [28, 29]. Utilizing a different configuration, where a vacuum is applied to obtain a pressure differential to facilitate the permeation rather than a sweep gas, allows for pure hydrogen to be obtained in the permeate. Thus, there is a need to identify a catalyst that performs the ammonia cracking reaction within the narrow range of reactivity, between 350°C and 450°C, as that would allow for PMR systems with ammonia cracking further explored and eventually become operational.

Low‐temperature ammonia cracking (<500°C) is currently relatively unexplored, and literature examples showcase the need for fine tuning of the catalyst. For example, Ru‐based heterogenous catalysts are often employed for ammonia cracking reactions, but typical and commercial catalysts require temperatures above 600°C [30, 31, 32, 33]. Tuning the catalyst support enhances the catalytic performance at low temperatures, with carbon‐based supports greatly improving the catalytic efficiency. Carbon‐based catalysts, however, are not stable to contaminate in the gas feed nor are they radiolytically stable [34]. In contrast, *γ*‐Al_2_O_3_ or CeO_2_ are also highly active support materials while also being stable to contaminants [35, 36]. The incorporation of additional metals, like potassium, to form a 4%Ru/12%K/Al_2_O_3_ catalyst, has shown catalytic activity improvements, allowing for further improvements in the yield of hydrogen [37]. An additional promoter, yttrium to form a 3%Ru/1%Y/12%K/*γ*‐Al_2_O_3_ catalyst (further referred to as RuKY), has also been shown to further improve the catalytic properties of the catalyst and is active at temperatures significantly lower than previously reported ammonia cracking catalysts [3, 38]. However, even RuKY catalysts can be further optimized as the precursors utilized generated catalysts with varying degrees of ammonia cracking efficiency [39]. Combined, the aforementioned optimizations have yielded catalysts with exceptional ammonia cracking efficiency at low temperatures.

**FIGURE 1:**
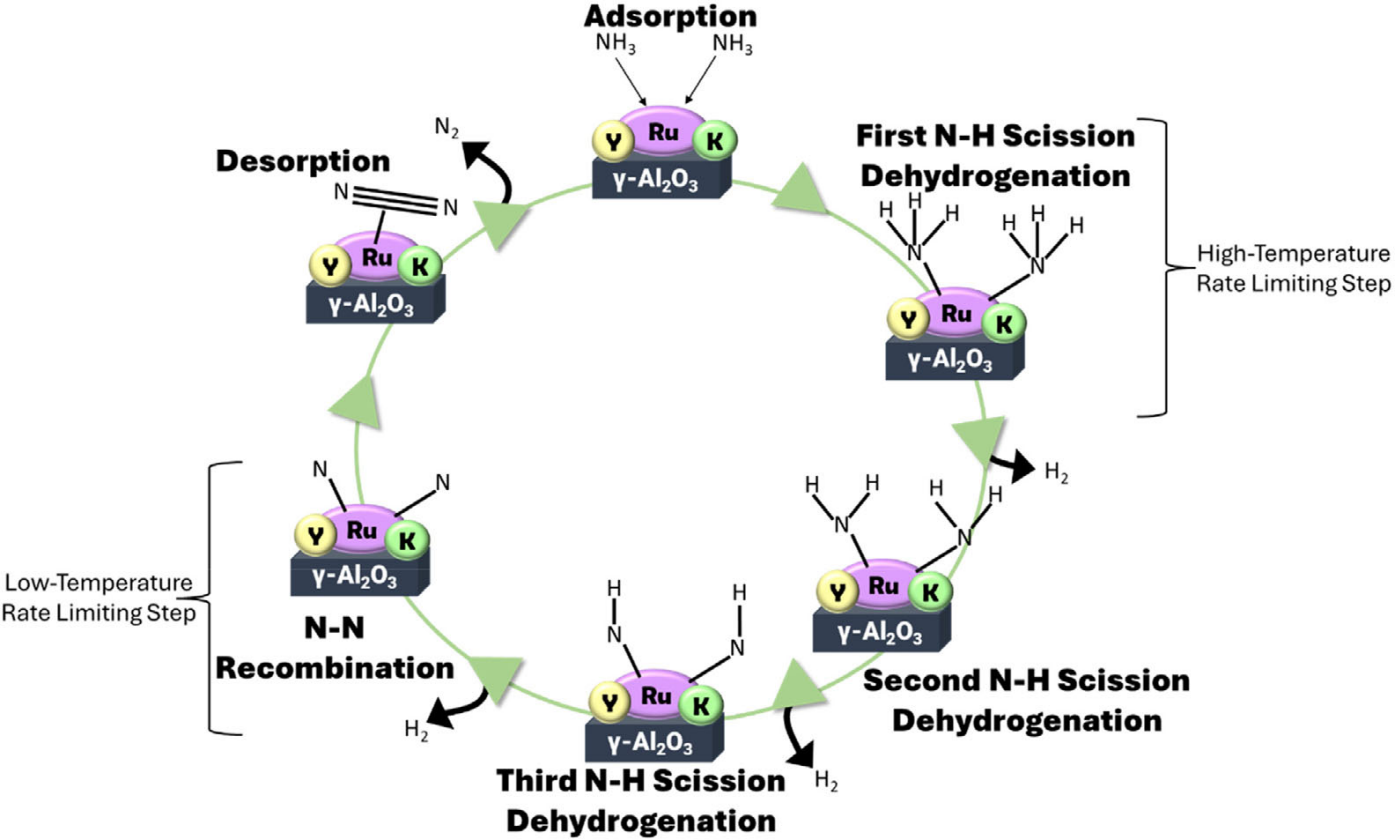
Proposed ammonia decomposition (“ammonia cracking”) mechanism.

While permeation membrane reactors are beneficial for all hydrogen carriers, fusion reactors will experience all common isotopologues of ammonia (NH_3_, ND_3_, NT_3_). Therefore, it is of a great importance to know the relevant rate‐limiting steps of ammonia cracking, which is theorized to be either a N–N recombination step or three N–H scission steps (Figure 1) [40, 41]. It has previously been reported that at lower temperatures, the N–N recombination step is the rate‐limiting step of the reaction [42, 43, 44, 45]. However, upon increasing the temperature, the rate‐limiting step becomes one of the three N–H scission steps [46, 47]. This is of great importance in the setting of the permeation membrane reactor as the rate‐limiting step of N–H scission would be impacted by isotopic changes in the hydrogen isotope of ammonia.

Herein, we report a trimetallic ruthenium‐based catalyst, 3%Ru/12%K/1%Y/Al_2_O_3_, for improved performance in the ammonia cracking reaction and its use in a permeation membrane reactor (PMR). The RuKY displays extraordinary ammonia cracking efficiency (>93.9% at 400°C). The activation energies of the RuKY catalyst were independent of isotopologue at PMR‐relevant temperatures (<500°C), indicating the rate‐limiting step does not involve the N—H bond. However, at higher temperatures, there is a difference in activation energies. Additionally, at 450°C, the RuKY catalyst achieved high conversion rates with a hydrogen production of 1.12 mol/g_cat_·h and 62.6% conversion and a deuterium production rate of 0.87 mol/g_cat_·h and 48.7% conversion rivaling other Ru‐based catalysts reported [48, 49]. When used within a PMR, a <1 ppm ammonia effluent was achieved, completely eliminating any further purification and separation processes. This allows for, to the best of our knowledge, the coldest temperatures for quantitative ammonia removal and allows for additional reactions to be utilized with permeation membrane reactors rather than the traditional gas‐water shift reaction, allowing for more applications of permeation membrane reactor.

## 2 Experimental

### 2.1 Catalyst Synthesis

Ruthenium (III) nitrosyl nitrate (Ru(NO_3_)_3_NO, ThermoFisher Scientific, lot# Z20J027), yttrium (III) nitrate hexahydrate (Y(NO_3_)_3_·6H_2_O, ThermoFisher Scientific, 99.9%), and potassium nitrate (KNO_3_, ThermoFisher Scientific, 99%) were purchased and utilized without further purification. Al_2_O_3_ (*γ*‐Al_2_O_3_, Sasol, lot# TK2347) was purchased and utilized without further purification. The precursors were measured to obtain a final weight composition of 3%Ru/1%Y/12%K/Al_2_O_3_ (denoted as RuKY). These precursors were dissolved in 5 mL of DI H_2_O and the alumina pellets were measured and placed into a crucible. The solution was added dropwise to the alumina pellets, while the pellets were being manually stirred. After the solution was added, an additional 1 mL of DI H_2_O was added to the container containing the metal solution to ensure that the solution was transferred. The catalyst was then dried overnight at 80°C and then calcined at 550°C (heating rate of 20°C/min) for 3 h.

### 2.2 Ammonia Cracking Reaction: Fixed Bed Reactor

0.5 g of catalyst was added to the reactor vessel and purged for 30 min with Ar (40 mL/min). The vessel was then heated to 450°C, and the catalyst was activated under an atmosphere of Ar (40 mL/min) and H_2_ (10 mL/min) for 4 h. After the activation was completed, the system was purged for 30 min with Ar to remove residual H_2_. After purging, ammonia was introduced into the system at 1 atm pressure and at the desired flow rate.

### 2.3 Conversion Calculation

(1) $$\text{\%}\text{conversion}=\frac{\text{ammonia converted}}{\text{initial ammonia}}=\frac{2p_{N_{2}}}{p_{NH_{3}}+2p_{N_{2}}}*100$$

Equation (1) shows the conversion calculation for the ammonia cracking reaction.

The conversion rate was calculated from the pressure registered from the RGA signals (MKS Cirrus 2). Then, the pressure of nitrogen was divided by the summation of ammonia and nitrogen and then multiplied by 100 for the conversion rate. The pressures were adjusted for the stoichiometric conversion of ammonia, and thus, the nitrogen signal was multiplied by 2.

### 2.4 Ammonia Cracking Reaction: Permeation Membrane Reactor

The PdAg tubing had a length of 4.2 cm, a diameter of 0.3 cm, and a wall thickness of 127 µm. The reactor was filled with 4.3 g of catalyst and then heated to 450°C. The catalyst was activated under the same conditions as previously mentioned. A vacuum pump (Varian Tri Scroll Vacuum Pump) was connected to the shell of the reactor to allow for permeation of H_2_. A NH_3_ Mettler‐Toledo GmbH Tunable Diode Laser, which has a limit of detection and resolution of 1 ppm, was utilized for analysis of low concentration ammonia in the effluent stream.

### 2.5 Raman Spectroscopy

Raman spectroscopic measurements were performed with a Horiba iHR320 spectrometer (0.318 m; 600 lines/mm grating) equipped with a Syncerity cooled CCD detector (–60°C). The spectra were collected in the 180° backscattering geometry using 532 nm excitation (532 nm 1500 mW Green DPSS, Civil Laser Supplier, NaKu Technology Co., Ltd). The commercially available silver‐lined, glass capillary has an inner diameter of 1 mm and a length of 500 mm (Guiding Photonics).

### 2.6 Scanning Electron Microscopy (SEM)

The SEM was obtained on a Hitachi SU8200. The voltage was set to 20 kV, and the accelerating voltage was set to 10 keV.

### 2.7 Transmission Electron Microscopy (TEM)

Bright‐field transmission electron microscopy (TEM) micrographs were acquired from particles deposited onto a lacey carbon‐coated grid. Imaging was performed using a JEOL JEM 2100F operated at an acceleration voltage of 200 kV. TEM is particularly well suited for resolving the morphology and fine structural features of these particles.

### 2.8 Wide Angle X‐Ray Scattering (WAXS)

Wide angle X‐ray scattering (WAXS) measurements were collected on a Xeuss 3.0 (Xenocs, France) equipped with a D2+ MetalJet X‐ray source (Ga K*α*, 9.2 keV, *λ* = 1.3414 Å) to analyze the structure of nickel‐based catalysts. Catalyst powders were formed into conformal layers with a nominal thickness of 0.5 mm and sealed between Kapton windows. The powders were aligned perpendicular to the direction of the incident beam (transmission mode), measured for 180 s at a sample‐to‐detector distance of 47 following calibration with a AgBeh standard. 2D images of the scattering patterns were collected on a Eiger 2R 4 M hybrid photon counting detector with a pixel dimension of 75 × 75 μm^2^ (Dectris, Switzerland). Azimuthal averages were reduced from the 2D WAXS images and plotted in the form of intensity versus scattering vector to yield a Q range of a 0.6–5.0 Å^−1^ (corresponding to a 2*Θ* range of 7.4–64.4° when converted to a copper source).

## 3 Results and Discussions

**FIGURE 2:**
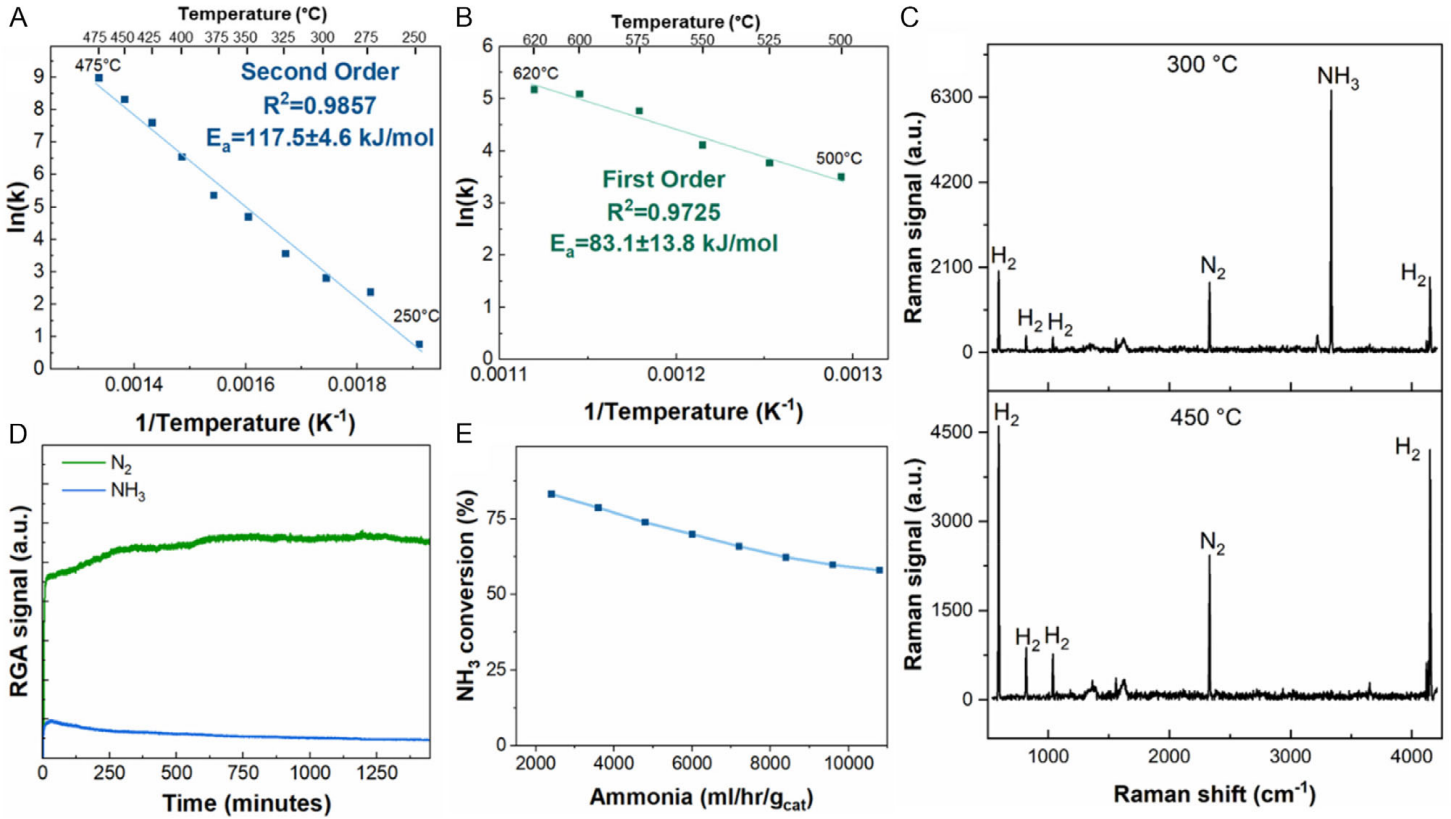
RuKY reacting with NH_3_. (A) Arrhenius plot with NH_3_ as the reactant from 250 to 475°C, (B) Arrhenius plot with NH_3_ as the reactant from 500°C to 620°C, (C) Raman spectroscopy of the gas effluent showcasing the difference in peaks between a low converting reaction and high converting reaction (conditions: 10% NH_3_ flow), (D) 24 stability of the catalyst under a 10% NH_3_ flow, and (E) increasing 100% ammonia flow rates and its effect on ammonia conversion at 450°C.

The RuKY catalysts were synthesized from nitrate salt precursors and low‐chloride alumina pellets to eliminate sources of chlorides and other halogens from the catalysts which may either damage the Pd alloy reactor or potentially react with radioactive species. The RuKY catalyst is extremely active in the relevant temperature range of a Pd membrane (conversion data and reaction rates shown in Figure 2), which is between 275°C and 500°C, though we note that Pd alloy permeators are usually run between 325°C and 450°C to maximize lifetime. Due to the PMR having a relatively low operating temperature range in comparison to ammonia cracking reactions, it was necessary to determine the reaction kinetics throughout the operating temperature range. It is known that the rate‐limiting step of ammonia cracking over Ru is temperature dependent [50]. Below 475°C, the reaction exhibits second‐order kinetics with respect to ammonia (Figure 2A), but first‐order kinetics above 475°C (Figure 2B). Additionally, the activation energy decreases from 117.5 kJ/mol to 83 kJ/mol, indicating that there is a change in the rate‐limiting step at ~475°C. We hypothesize that at low temperatures, the rate‐limiting step is the recombination of two nitrogen atoms to form N_2_, but at higher temperatures, the rate‐limiting step becomes the scission of the N—H bond (scheme shown in Figure 1). Raman spectroscopy was used to confirm the conversion of each species within the reaction to both confirm the RGA measurements and confirm the absence of side product formation (Figure 2C). Raman spectroscopy allows for more resolution between ammonia and its isotopologues than mass spectrometry does, which means that the ammonia signals can be better resolved from each other and from water. We note that the rate‐limiting step of N—H bond scission is extremely relevant utilized in the context of nuclear and fusion energy as a mixture of isotopologues would have large effects on the performance of the PMR. The stability of the RuKY catalyst, both in respect to reaction with ammonia and potential reaction with the PMR membrane, is crucial for its adoption as an ammonia cracking catalyst. To confirm the midterm stability of the RuKY, the catalyst was exposed to ammonia for 24 h (Figure 2D), with no detrimental effect to the catalyst.

**FIGURE 3:**
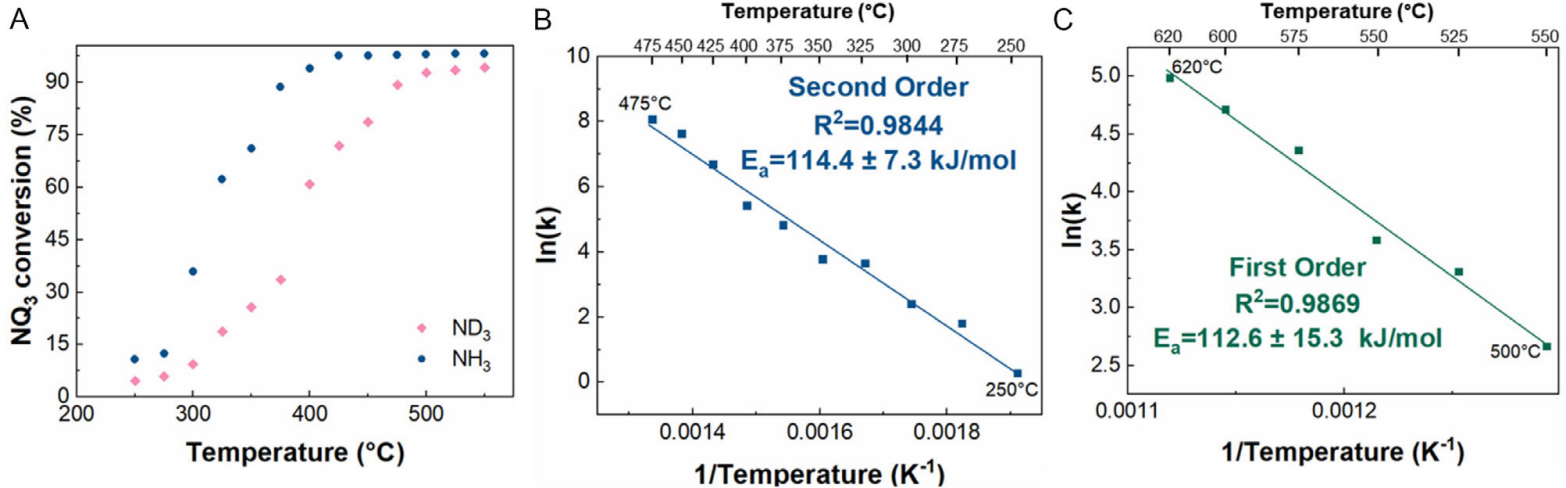
RuKY reacting with ND_3_. (A) Comparison of NH_3_ and ND_3_ (where NQ_3_ is used to mean either NH_3_ or ND_3_ depending on the isotopologue utilized) reacting with 10%NH_3_ flow rates, (B) Arrhenius plot with ND_3_ as the reactant from 250°C to 475°C, and (C) Arrhenius plot with ND_3_ as the reactant from 500°C to 620°C.

Hydrogen‐containing molecules are often more reactive than deuterium isotopologues, which is referred to as the kinetic isotope effect (KIE) [51, 52]. To determine the KIE of the ammonia cracking reaction over the RuKY catalyst, the reaction kinetic experiments were repeated with ND_3_ and D_2_, replacing NH_3_, and H_2_ (conversion as a function of temperature shown in Figure 3A). Across all temperatures measured, the RuKY was more active toward the hydrogen isotopologues than the deuterium isotopologues. Arrhenius plots for the ammonia cracking of ND_3_ are shown in Figure 3B (low temperature) and 3C (high temperature). Below 475°C, the reaction remains 2^nd^ order and has an activation energy of 114 kJ/mol, which is nearly identical to the activation energy of the hydrogen isotope (NH_3_). The isotope independence of the activation energy confirms our earlier hypothesis that the rate‐limiting step of the low‐temperature reaction does not involve the H/D species (Figure 1), and likely is the N–N recombination. In contrast, activation energy above 475°C is also 113 kJ/mol, a 36% increase over the hydrogen isotope, confirming that the first‐order rate‐limiting step involves the scission of the N—H/D bond.

Any observed difference in low‐temperature reactivity between NH_3_ and ND_3_ is most likely due to the differences in the adsorption of NH_3_ and ND_3_ on the catalyst. The greater adsorption of NH_3_ creates an observed kinetic isotope effect at all tested temperatures. However, we note that the kinetic isotope effect (KIE) (k_H_/k_D_) is noticeably different between the two temperature ranges tested. The lower temperature <475°C have no pattern in the KIE that can be observed. However, at higher temperatures (>500°C), the KIE decreases as the temperature increases. While the rate constants exhibit a KIE, the fact that the activation energies remain similar when the reaction is second order indicates that the rate‐limiting step is indeed N–N recombination and that the difference in reactivity is due to the difference in concentration of ammonia on the catalyst surface. This is valuable in understanding how the catalyst will perform in the permeation membrane reactor but has not reached high enough conversions to circumvent NO_*x*_ generation necessitating further studies in a PdAg permeation tubing.

**FIGURE 4:**
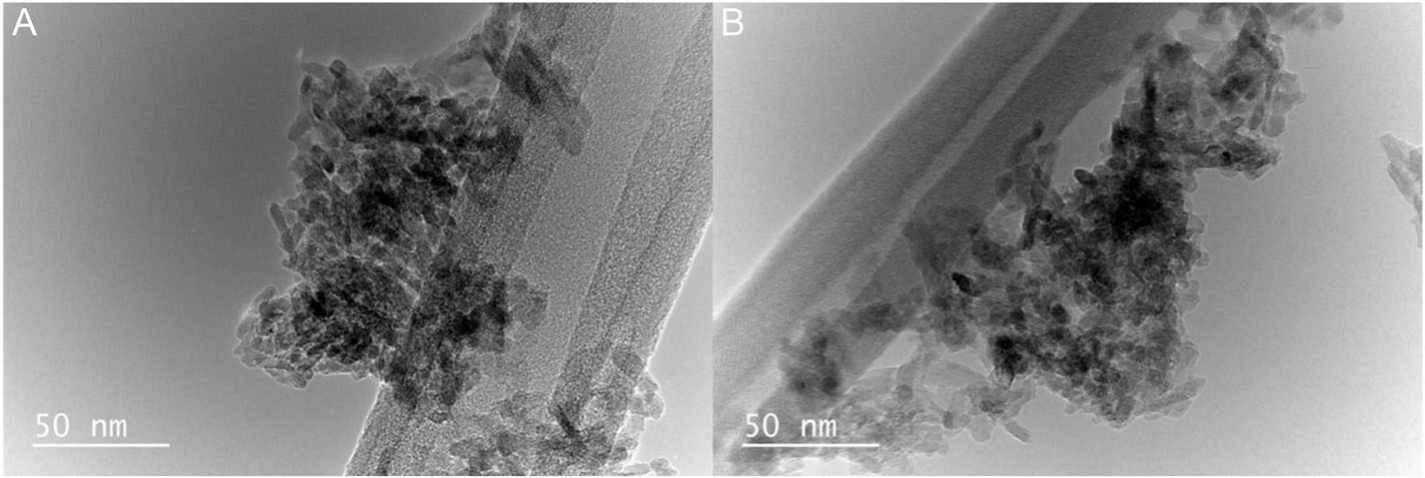
Transmission electron microscopy images: (A) before reaction and (B) after reaction.

Transmission electron microscopy (TEM) and scanning electron microscopy (SEM) were utilized to understand the structure and morphological changes of the catalyst before and after the reaction. Figure 4 shows the TEM images before and after the reaction, where the catalyst has nominal changes over the course of the reaction, indicating that the catalyst does not have agglomeration or phase changes occurring. SEM images are in the Supporting Information, where the before (Figure S1) shows that there are cube‐like structures in the material. The cube‐like structures are no longer present in the SEM image after the reaction (Figure S2), where there are now spear‐like structures, indicating that there is an increase of surface area on the surface of the catalyst. Wide‐angle X‐ray scattering (WAXS) was collected to determine if there are phase changes occurring in the material. The WAXS spectrum before (Figure S3) and after (Figure S4) provides similar signals, with the primary difference being the introduction of activation of the ruthenium metal adding new peaks. These three characterization techniques help to indicate that the catalyst has nominal changes over the course of the reaction, allowing for the catalyst to be utilized in a variety of applications.

The RuKY catalyst was packed into a Pd‐Ag tube as a PMR system to characterize the synergistic effects of in situ hydrogen removal (diagram of the system shown in Figure 4A). The synergistic ammonia cracking reaction was characterized from 350°C to 450°C, which is the safe operating temperature of the PdAg alloy. Importantly, this temperature range does not include temperatures where the rate‐limiting step is in the N–H scission step, which is beneficial as isotopologues should not induce a kinetic isotope effect within the permeation reactor. Hydrogen removal can be toggled off and on by modulating the vacuum on the permeate side of the PdAg tube, which allows for direct quantification of the PMR's effect on the NH_3_ cracking reaction. The NH_3_ conversion as a function of temperature and flow rate, with and without vacuum, is shown in Figure 4B,C respectively. As expected, without in situ hydrogen removal, the conversion of NH_3_ only reached ~99% above 450°C and at the lowest measured flow rate. Below 450°C, the efficiency of the reaction was poor at all flow rates. In contrast, nearly quantitative (>99%) NH_3_ conversion was achieved above >375°C when hydrogen was removed via the PMR across a wide range of flow rates. This reactivity was also demonstrated to be stable for 15 h, allowing for use of the system without deactivation of either the catalyst or membrane (Figure S8). We note that as the H_2_ permeability of Pd‐Ag increases with temperature, the PMR efficiency increases more than might be expected with temperature due to the synergy between the RuKY catalyst and PMR. As the conversion approaches 100%, the shifting of the reaction equilibrium due to Le Chatelier's principle becomes significant, enabling conversions far beyond those possible in a simple packed bed reactor system. Utilizing a PMR instead of a simple packed bed enables higher reagent flows, greater maximal conversion, while simultaneously producing pure hydrogen.

**FIGURE 5:**
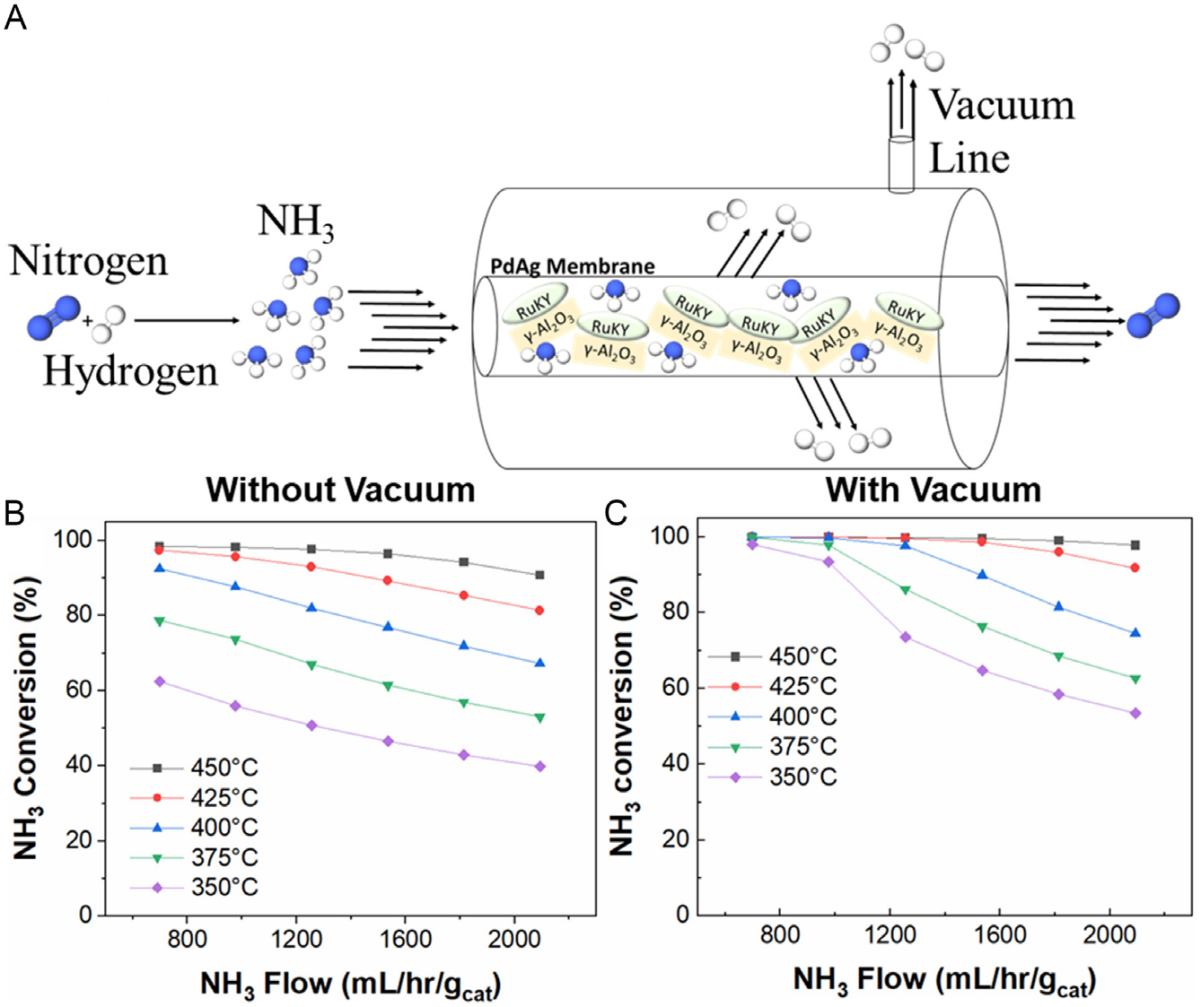
(A) Conversion of ammonia into nitrogen and hydrogen utilizing the PdAg permeation membrane reactor (C) with vacuum and (B) without vacuum.

The isotope dependence of the ammonia cracking reaction of the RuKY‐packed PMR was characterized via ND_3_ and D_2_. The NH_3_ and ND_3_ (generalized as NQ_3_) conversions of RuKY‐packed PMR as a function of temperature (400°C–450°C) and flow rate are shown in Figure 5. We expect that the efficiency of the ammonia cracking reaction should be decreased relative to hydrogen isotopologues, as the PdAg alloy exhibits a lower permeability to D_2_ than H_2_ and that the RuKY exhibits lower reactivity, as discussed before. Without H_2_ or D_2_ removal (Figure 5B), the efficiency of the NQ_3_ cracking reaction follows the general trend of the simple packed bed reactor discussed previously. At all measured temperatures, the reactivity of NH_3_ is greater than ND_3_. With H_2_ or D_2_ removal (Figure 5A), the efficiency of the NQ_3_ cracking reaction is significantly enhanced. However, at all measured temperatures and flow rates, the ND_3_ conversion is lower than the NH_3_ equivalent. Despite this, >99% ND_3_ conversion is still readily achieved at >425°C and at lower flow rates, highlighting the enhancement made possible by the PMR. While the ammonia cracking efficiency is unprecedentedly high, the reactivity at higher flow rates is limited due to the Q_2_ permeation through the membrane. Further advancements in permeation membranes would allow for higher conversions of ammonia decomposition at these higher flow rates, allowing for a more efficient process.

**FIGURE 6:**
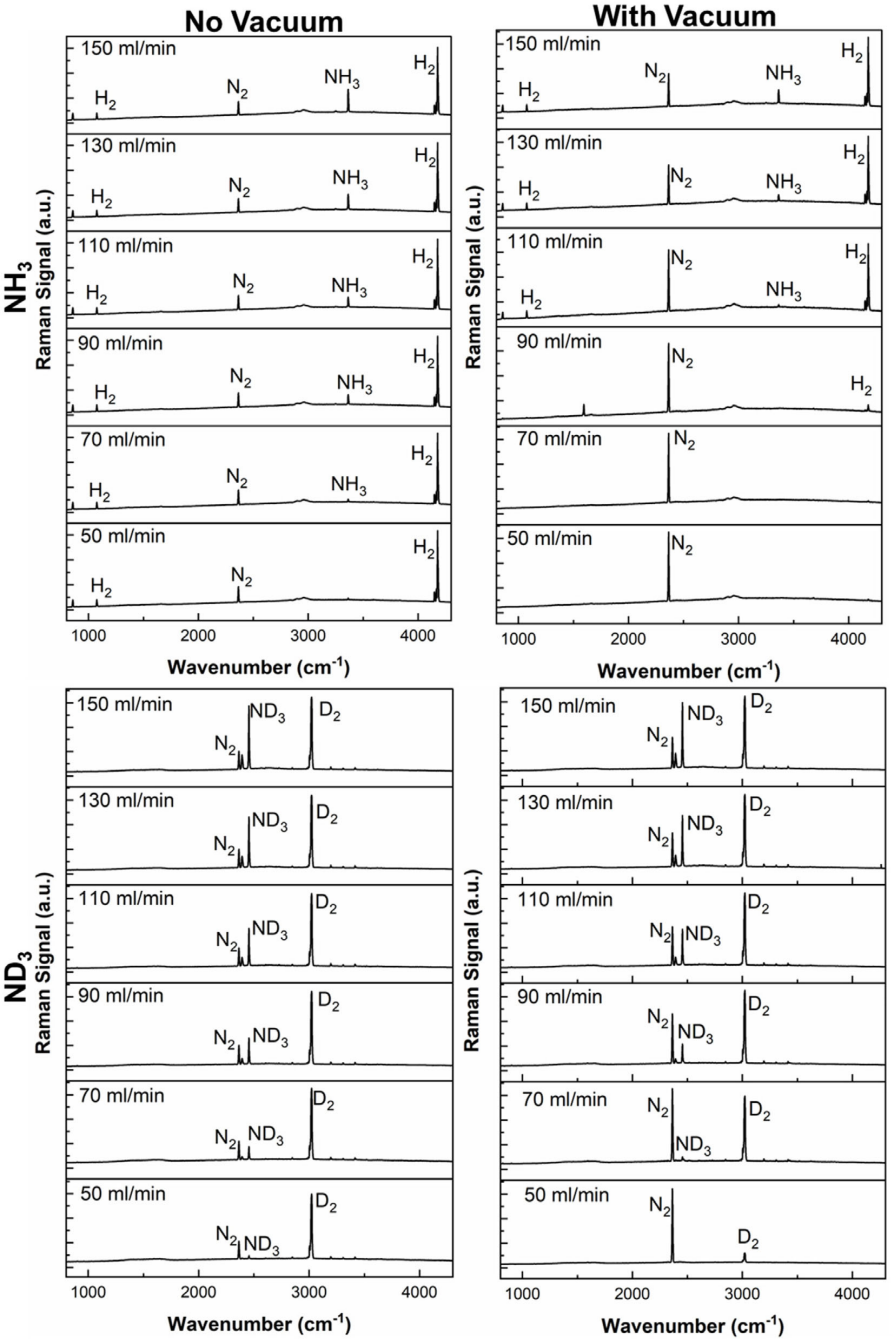
Comparison of reactivity of NH_3_ and ND_3_ utilizing the RuKY catalyst utilizing Raman spectroscopy at 450°C.

**FIGURE 7:**
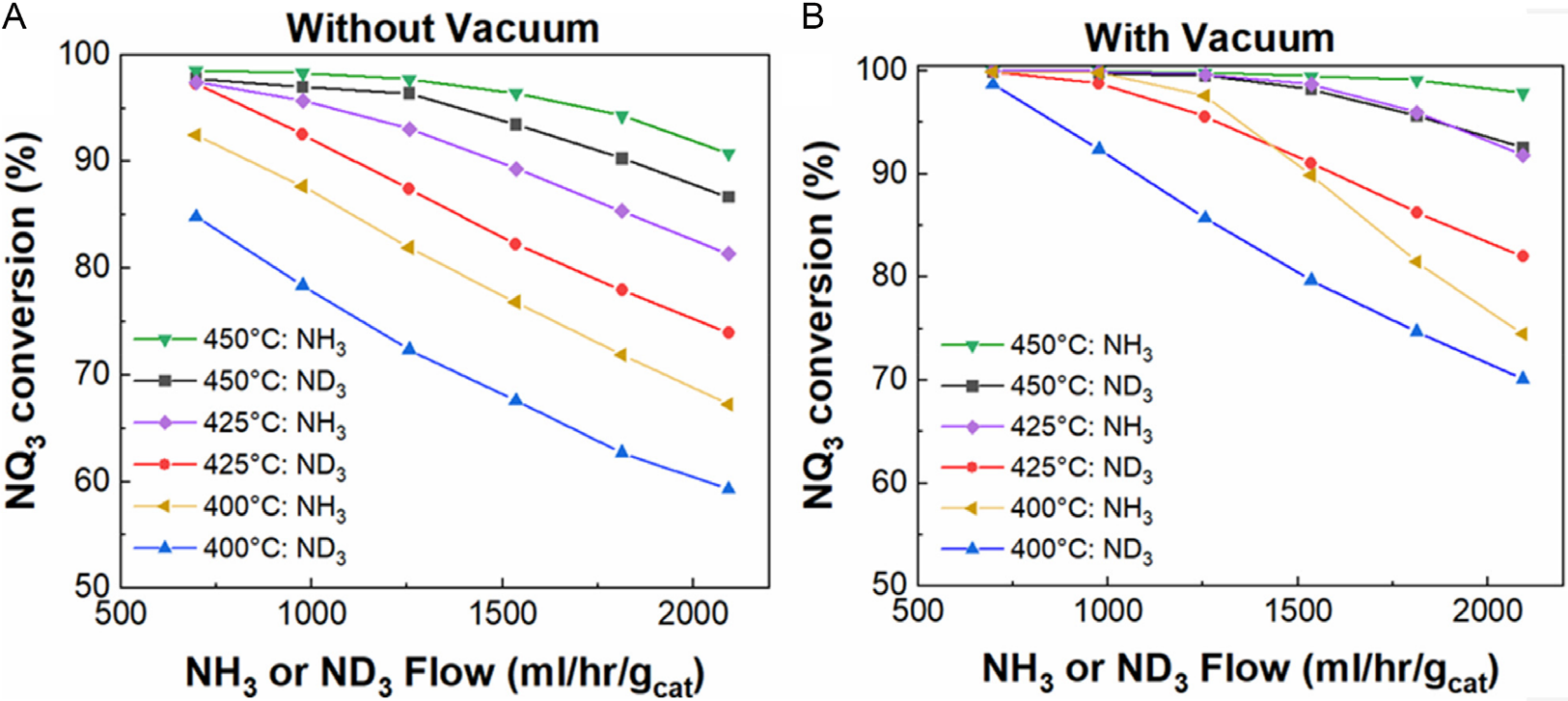
Conversion of ammonia (both NH_3_ and ND_3_, denoted as NQ_3_ in the graph) into nitrogen and hydrogen/deuterium utilizing the PdAg permeation membrane reactor (A) with vacuum and (B) without vacuum.

While mass spectrometry is helpful for identifying the conversion of the reaction, it is not sensitive enough for identifying minute changes in the reaction system, especially of H_2_ concentration. In contrast, Raman spectroscopy can identify small changes of all reagent and products within the PMR system, which allows for accurate measurement of the PMR breakthrough conditions (Figure 6). Therefore, Raman spectroscopy was used to monitor NQ_3_ conversion and Q_2_ extraction of both NH_3_ and ND_3_, with and without vacuum‐induced permeation. From Figure 7, it can be seen that the breakthrough of the system occurs at higher flow rates for NH_3_ compared to ND_3_, both with and without permeation through the membrane. For NH_3_, when no vacuum is utilized, there is a strong H_2_ peak at 4192 cm^−1^ and a large N_2_ peak at 2363 cm^−1^, indicating that hydrogen is being synthesized. At all flow rates without vacuum, the NH_3_ peak at 3365 cm^−1^ remains present. Equivalently, ND_3_ (2560 cm^−1^) is always present in the deuterium system without vacuum, while D_2_ (3022 cm^−1^) and N_2_ are synthesized. When vacuum is applied to the PMR system, inducing permeation through the walls, the conversion of NH_3_ or ND_3_ is greatly enhanced, and the H_2_ and D_2_ signals are reduced or eliminated entirely. Incredible, a pure N_2_ product stream was achieved at 70 mL/min of NH_3_, indicating that not only was the NH_3_ fully converted, but that the entirety of H_2_ was removed.

**FIGURE 8:**
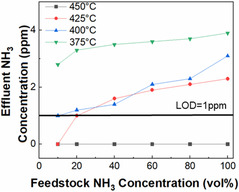
NH_3_ cracking efficiency of a PMR with vacuum as a function of temperature and NH_3_ feedstock concentration as measured by a long pathlength tunable diode laser. Values below the limit of detection are displayed as 0 ppm. The ppm amounts for the reaction at 350°C was significantly higher and is shown in Figure S12 for clarity (conditions: 50 mL/min total flow, 4.3 g RuKY catalyst, 1 atm).

For many applications, such as for fuel cells in dense urban environments and fusion power, it is crucial that the concentration of vented NH_3_ be kept as low as possible. Both Raman spectroscopy and mass spectroscopy cannot resolve <1 ppm of NH_3_ due to their inherent limit of detection (LOD). In contrast, a tunable diode laser can resolve <1 ppm, due to the extremely long path length. Therefore, a tunable diode laser was utilized to analyze trace NH_3_ in the reactor effluent to confirm the ability of the PMR to achieve extremely trace NH_3_ effluent without additional separations (Figure 8). Above 400°C, the concentration of NH_3_ in the effluent is either below the LOD or trivial, highlighting the extraordinary efficiency of the PMR. Above 450°C, the ammonia concentration in the effluent is below the LOD at all flow rates. Without using a PMR, the effluent concentration never drops below 300,000 ppm, which is considerably greater concentration than with the PMR. Therefore, the combination of the low temperature RuKY catalyst and PMR enables industry‐relevant trace ammonia concentrations with no additional separations or purification.

## 4 Conclusion

In summary, we demonstrated the extraordinary ammonia cracking efficiency of a Pd alloy PMR containing a low temperature RuKY catalyst, which achieved >99.9999% conversion of ammonia to hydrogen and nitrogen in a single pass. This PMR was able to simultaneously generate pure streams of H_2_ and D_2_ from NH_3_ and ND_3_ respectively while achieving quantitative conversions. Importantly, the RuKY catalyst was shown to be stable under long reaction times. The reaction kinetics of the RuKY catalyst and combination with PMR was quantified through extensive kinetic testing, and it was found that the rate‐limiting step at low temperatures is second order attributable to N–N recombination, while it is first order at higher temperatures due to the N–H scission. This combined RuKY PMR system provides a path toward the utilization of ammonia as a fuel source, as the effluent NH_3_ concentration at relevant flow rates is significantly below the predicted requisite 99.6% conversion required for the maintenance of greenhouse gas emissions.

## Supporting Information

Additional supporting information can be found online in the Supporting Information section. **Supporting Fig. S1:** SEM image of the 3%Ru/1%Y/12%K/γ‐Al_2_O_3_ catalyst before reaction. **Supporting Fig. S2**: SEM image of the 3%Ru/1%Y/12%K/γ‐Al_2_O_3_ catalyst after reaction. **Supporting Fig. S3:** WAXS spectrum (where the *x*‐axis is converted to 2Θ with a copper laser source) of the 3%Ru/1%Y/12%K/γ‐Al_2_O_3_ catalyst before reaction. **Supporting Fig. S4:** WAXS spectrum (where the x‐axis is converted to 2Θ with a copper laser source) of the 3%Ru/1%Y/12%K/γ‐Al_2_O_3_ catalyst after reaction. **Supporting Fig. S5:** The conversion of NH_3_ at 620°C at various pure ammonia flow rates that were used to construct the rate plot. **Supporting Fig. S6:** The conversion of NH_3_ at 600°C at various pure ammonia flow rates that were used to construct the rate plot. **Supporting Fig. S7:** The conversion of NH_3_ at 575°C at various pure ammonia flow rates that were used to construct the rate plot. **Supporting Fig. S8:** The conversion of NH_3_ at 550°C at various pure ammonia flow rates that were used to construct the rate plot. **Supporting Fig. S9:** The conversion of NH_3_ at 525°C at various pure ammonia flow rates that were used to construct the rate plot. **Supporting Fig. S10:** The conversion of NH_3_ at 500°C at various pure ammonia flow rates that were used to construct the rate plot. **Supporting Fig. S11:** The conversion of NH_3_ at 475°C at various pure ammonia flow rates that were used to construct the rate plot. **Supporting Fig. S12:** The conversion of NH_3_ at 425°C at various pure ammonia flow rates that were used to construct the rate plot. **Supporting Fig. S13:** The conversion of NH_3_ at 400°C at various pure ammonia flow rates that were used to construct the rate plot. **Supporting Fig. S14:** The conversion of NH_3_ at 375°C at various pure ammonia flow rates that were used to construct the rate plot. **Supporting Fig. S15:** The conversion of NH_3_ at 350°C at various pure ammonia flow rates that were used to construct the rate plot. **Supporting Fig. S16:** The conversion of NH_3_ at 325°C at various pure ammonia flow rates that were used to construct the rate plot. **Supporting Fig. S17:** The conversion of NH_3_ at 300°C at various pure ammonia flow rates that were used to construct the rate plot. **Supporting Fig. S18:** The conversion of NH_3_ at 275°C at various pure ammonia flow rates that were used to construct the rate plot. **Supporting Fig. S19:** The conversion of NH_3_ at 250°C at various pure ammonia flow rates that were used to construct the rate plot. **Supporting Fig. S20:** The conversion of ND_3_ at 620°C at various pure ammonia flow rates that were used to construct the rate plot. **Supporting Fig. S21:** The conversion of ND_3_ at 600°C at various pure ammonia flow rates that were used to construct the rate plot. **Supporting Fig. S22:** The conversion of ND_3_ at 575°C at various pure ammonia flow rates that were used to construct the rate plot. **Supporting Fig. S23:** The conversion of ND_3_ at 550°C at various pure ammonia flow rates that were used to construct the rate plot. **Supporting Fig. S24:** The conversion of ND_3_ at 525°C at various pure ammonia flow rates that were used to construct the rate plot. **Supporting Fig. S25:** The conversion of ND_3_ at 500°C at various pure ammonia flow rates that were used to construct the rate plot. **Supporting Fig. S26:** The conversion of ND_3_ at 475°C at various pure ammonia flow rates that were used to construct the rate plot. **Supporting Fig. S27:** The conversion of ND_3_ at 450°C at various pure ammonia flow rates that were used to construct the rate plot. **Supporting Fig. S28:** The conversion of ND_3_ at 425°C at various pure ammonia flow rates that were used to construct the rate plot. **Supporting Fig. S29:** The conversion of ND_3_ at 400°C at various pure ammonia flow rates that were used to construct the rate plot. **Supporting Fig. S30:** The conversion of ND_3_ at 375°C at various pure ammonia flow rates that were used to construct the rate plot. **Supporting Fig. S31:** The conversion of ND_3_ at 350°C at various pure ammonia flow rates that were used to construct the rate plot. **Supporting Fig. S32:** The conversion of ND_3_ at 325°C at various pure ammonia flow rates that were used to construct the rate plot. **Supporting Fig. S33:** The conversion of ND_3_ at 300°C at various pure ammonia flow rates that were used to construct the rate plot. **Supporting Fig. S34:** The conversion of ND_3_ at 275°C at various pure ammonia flow rates that were used to construct the rate plot. **Supporting Fig. S35:** The conversion of ND_3_ at 250°C at various pure ammonia flow rates that were used to construct the rate plot. **Supporting Fig. S36:** The effect of hydrogen in the feedstock on the cracking of ammonia in the PMR. **Supporting Fig. S37:** Conversion of ammonia at varying inlet ammonia concentrations. **Supporting Fig. S38:** The effect of pressure on the conversion in a fixed bed reactor. **Supporting Fig. S39:** Stability of catalyst in permeation membrane reactor over 15 hours at 400°C. **Supporting Fig. S40:** Ammonia‐d_3_ conversion at varied flow rates. **Supporting Fig. S41:** The effect of deuterium in the feedstock on the cracking of ammonia‐d_3_ in the PMR. **Supporting Fig. S42:** NH_3_ cracking efficiency of a PMR at 350°C and NH_3_ feedstock concentration as measured by a long pathlength tunable diode laser. **Supporting Fig. S43:** NH_3_ cracking efficiency of a PMR as a function of temperature and NH_3_ feedstock concentration as measured by a long pathlength tunable diode laser with no vacuum being pulled. **Supporting Fig. S44:** Q_2_ recovery of the Permeation Membrane Reactor with the same conditions as in Figure 6B. **Supporting Table S1:** Phase and d spacing data corresponding to Figure S3. **Supporting Table S2:** Phase and d spacing data corresponding to Figure S4. **Supporting Table S3:** The turnover frequency and hydrogen productivity of the RuKY catalyst at 450°C. **Supporting Table S4:** The turnover frequency and hydrogen productivity of the RuKY catalyst at 620°C. **Supporting Table S5:** The turnover frequency and hydrogen productivity of the RuKY catalyst at 600°C. **Supporting Table S6:** The turnover frequency and hydrogen productivity of the RuKY catalyst at 575°C. **Supporting Table S7:** The turnover frequency and hydrogen productivity of the RuKY catalyst at 550°C. **Supporting Table S8:** The turnover frequency and hydrogen productivity of the RuKY catalyst at 525°C. **Supporting Table S9:** The turnover frequency and hydrogen productivity of the RuKY catalyst at 500°C. **Supporting Table S10:** The turnover frequency and hydrogen productivity of the RuKY catalyst at 475°C. **Supporting Table S11:** The turnover frequency and hydrogen productivity of the RuKY catalyst at 425°C. **Supporting Table S12:** The turnover frequency and hydrogen productivity of the RuKY catalyst at 400°C. **Supporting Table S13:** The turnover frequency and hydrogen productivity of the RuKY catalyst at 375°C. **Supporting Table S14:** The turnover frequency and hydrogen productivity of the RuKY catalyst at 350°C. **Supporting Table S15:** The turnover frequency and hydrogen productivity of the RuKY catalyst at 325°C. **Supporting Table S16:** The turnover frequency and hydrogen productivity of the RuKY catalyst at 300°C. **Supporting Table S17:** The turnover frequency and hydrogen productivity of the RuKY catalyst at 275°C. **Supporting Table S18:** The turnover frequency and hydrogen productivity of the RuKY catalyst at 250°C. **Supporting Table S19:** The turnover frequency and deuterium productivity of the RuKY catalyst at 620°C. **Supporting Table S20:** The turnover frequency and deuterium productivity of the RuKY catalyst at 600°C. **Supporting Table S21:** The turnover frequency and deuterium productivity of the RuKY catalyst at 575°C. **Supporting Table S22:** The turnover frequency and deuterium productivity of the RuKY catalyst at 550°C. **Supporting Table S23:** The turnover frequency and deuterium productivity of the RuKY catalyst at 525°C. **Supporting Table S24:** The turnover frequency and deuterium productivity of the RuKY catalyst at 500°C. **Supporting Table S25:** The turnover frequency and deuterium productivity of the RuKY catalyst at 475°C. **Supporting Table S25:** The turnover frequency and deuterium productivity of the RuKY catalyst at 450°C. **Supporting Table S26:** The turnover frequency and deuterium productivity of the RuKY catalyst at 425°C. **Supporting Table S27:** The turnover frequency and deuterium productivity of the RuKY catalyst at 400°C. **Supporting Table S28:** The turnover frequency and deuterium productivity of the RuKY catalyst at 375°C. **Supporting Table S29:** The turnover frequency and deuterium productivity of the RuKY catalyst at 350°C. **Supporting Table S30:** The turnover frequency and deuterium productivity of the RuKY catalyst at 325°C. **Supporting Table S31:** The turnover frequency and deuterium productivity of the RuKY catalyst at 300°C. **Supporting Table S32:** The turnover frequency and deuterium productivity of the RuKY catalyst at 275°C. **Supporting Table S32:** The turnover frequency and deuterium productivity of the RuKY catalyst at 250°C. **Supporting Table S33:** Second order rate constants for the RuKY catalyst for both NH_3_ and ND_3_ as the reactant. **Supporting Table S34:** First order rate constants for the RuKY catalyst for both NH_3_ and ND_3_ as the reactant.

## Funding

This study was supported by National Nuclear Security Administration and U.S. Department of Energy (89303321CEM000080).

## Conflicts of Interest

The authors declare no conflicts of interest.

## Supporting information

Supplementary Material

## Data Availability

The data that support the findings of this study are available from the corresponding author upon reasonable request.
