# Supplementary material for: RuKY Catalyst‐Packed Permeation Membrane for Quantitative Ammonia and d3‐Ammonia Dehydrogenation to Ultrapure Hydrogen
Source: ChemistryOpen. 2026 Jan 26;15(1):e202500480. doi: 10.1002/open.202500480 (PMC12835550; doi:10.1002/open.202500480)
Supplement: Supplementary file 1 — Supplementary Material [file OPEN-15-e202500480-s001.pdf]

Supporting Information

RuKY Catalyst-Packed Permeation Membrane for Quantitative Ammonia and d3-Ammonia Dehydrogenation to Ultrapure Hydrogen

Christopher J. Koch[†], Jennifer Naglic[†], John T. Kelly[†], Logan Kearney[‡], José D. Arregui-Mena[•], Jochen Lauterbach[Ψ], Lucas M. Angelette[†], Tyler Guin[†,*]

Contents

Scanning Electron Microscopy (SEM) 2

Wide Angle X-Ray Scattering (WAXS) 4

Kinetic Isotope Effect 8

Additional Permeation Membrane Data 31

Scanning Electron Microscopy (SEM)

The morphology of the catalyst was analyzed before (Figure S1) and after (Figure S2) reaction through Scanning Electron Microscopy (SEM). There is a clear morphology change over the course of the reaction, where cubic structures in the catalyst before reaction are converted to spears in the catalyst after, increasing surface area of the catalyst.

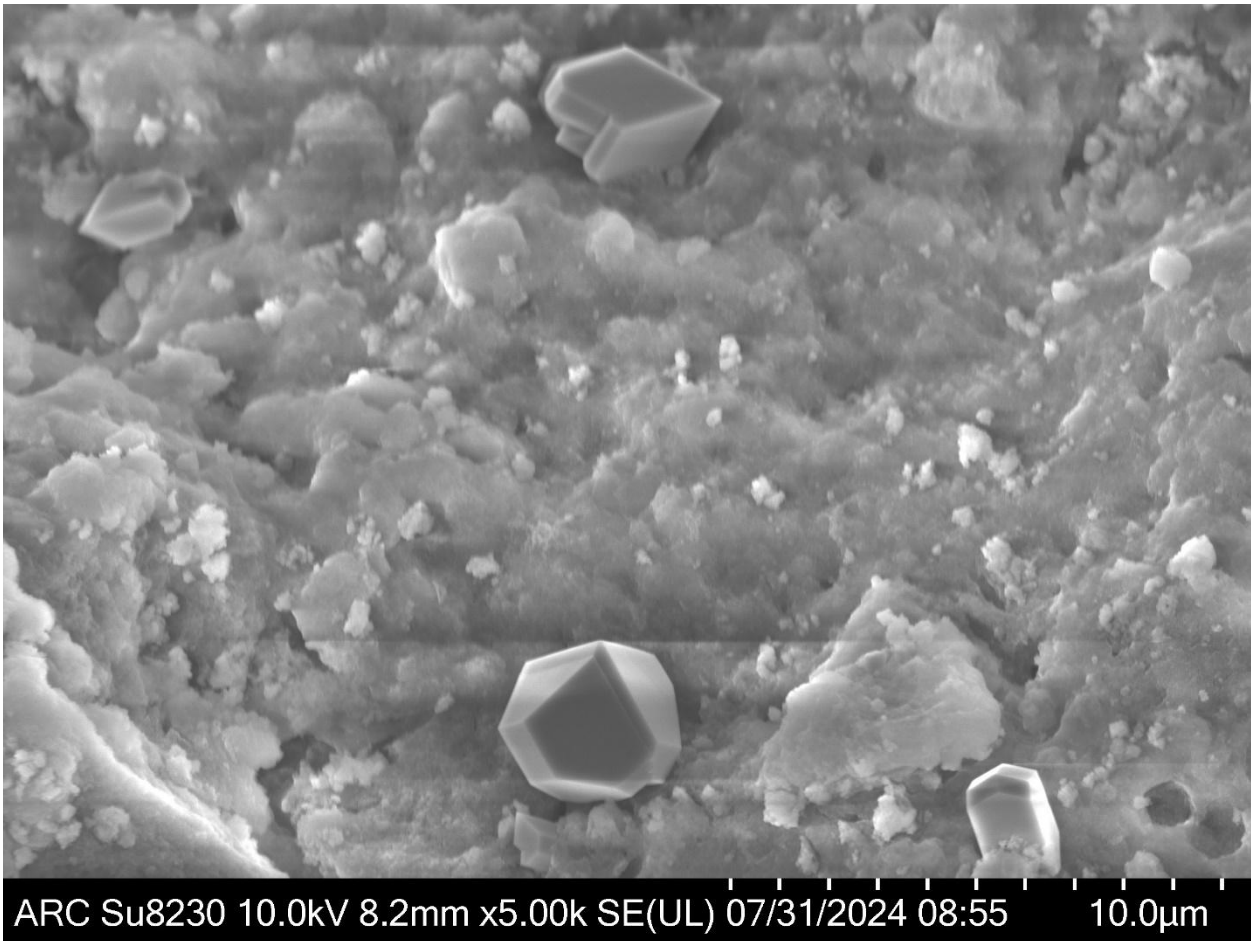

Figure S1. SEM image of the 3%Ru/1%Y/12%K/γ-Al_2O_3 catalyst before reaction.

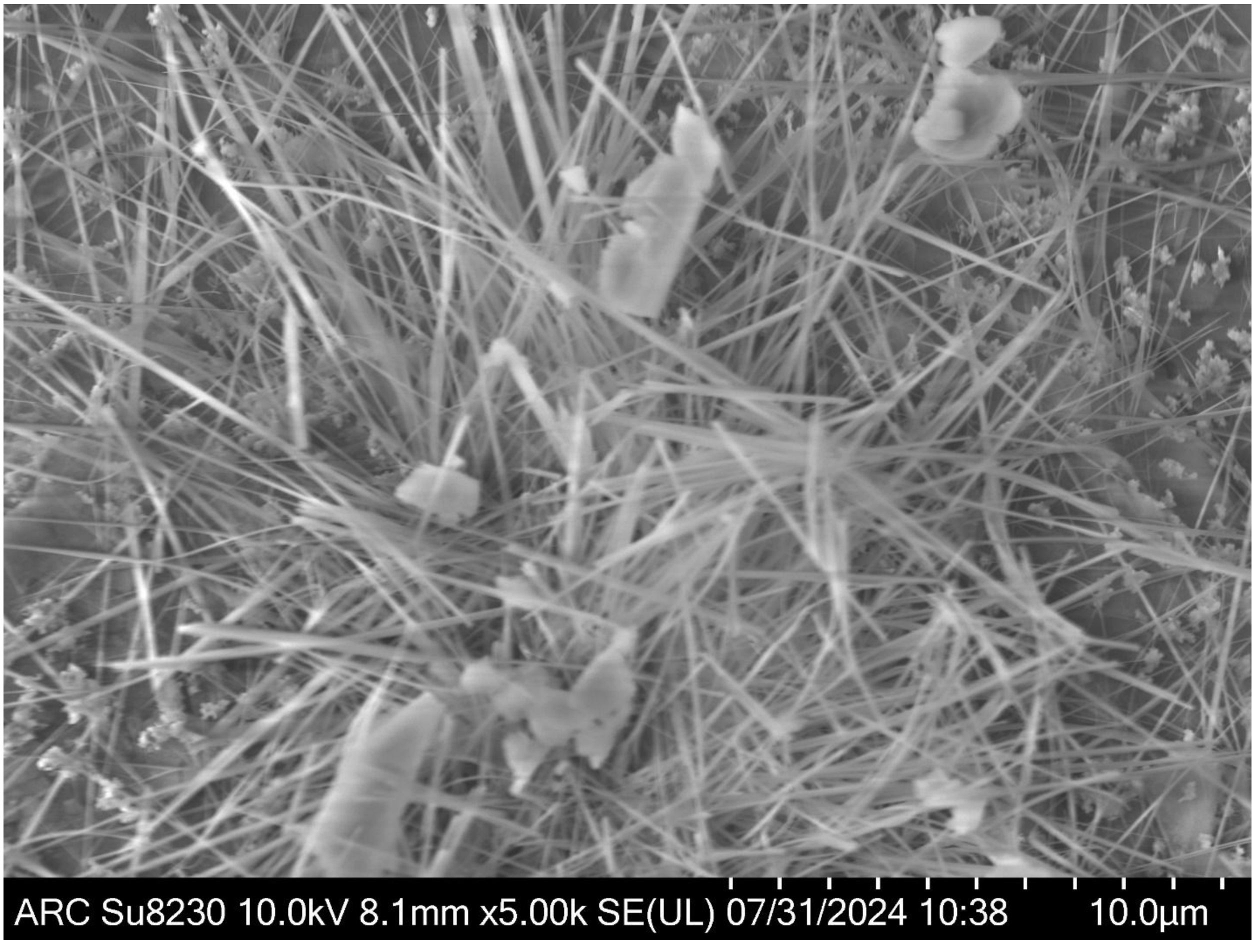

Figure S2. SEM image of the 3%Ru/1%Y/12%K/γ-Al_2O_3 catalyst after reaction.

Wide Angle X-Ray Scattering (WAXS)

Wide Angle X-Ray Scattering was utilized to obtain XRD patterns. The patterns before and after reaction are consistent with major differences between the two spectra being the activation of the ruthenium metal (from RuO2 to Ru) and a support phase change (from $K_{0.33}Al_3O_{4.67}$ to $K_{1.15}Al_{10.95}O_{17}$).

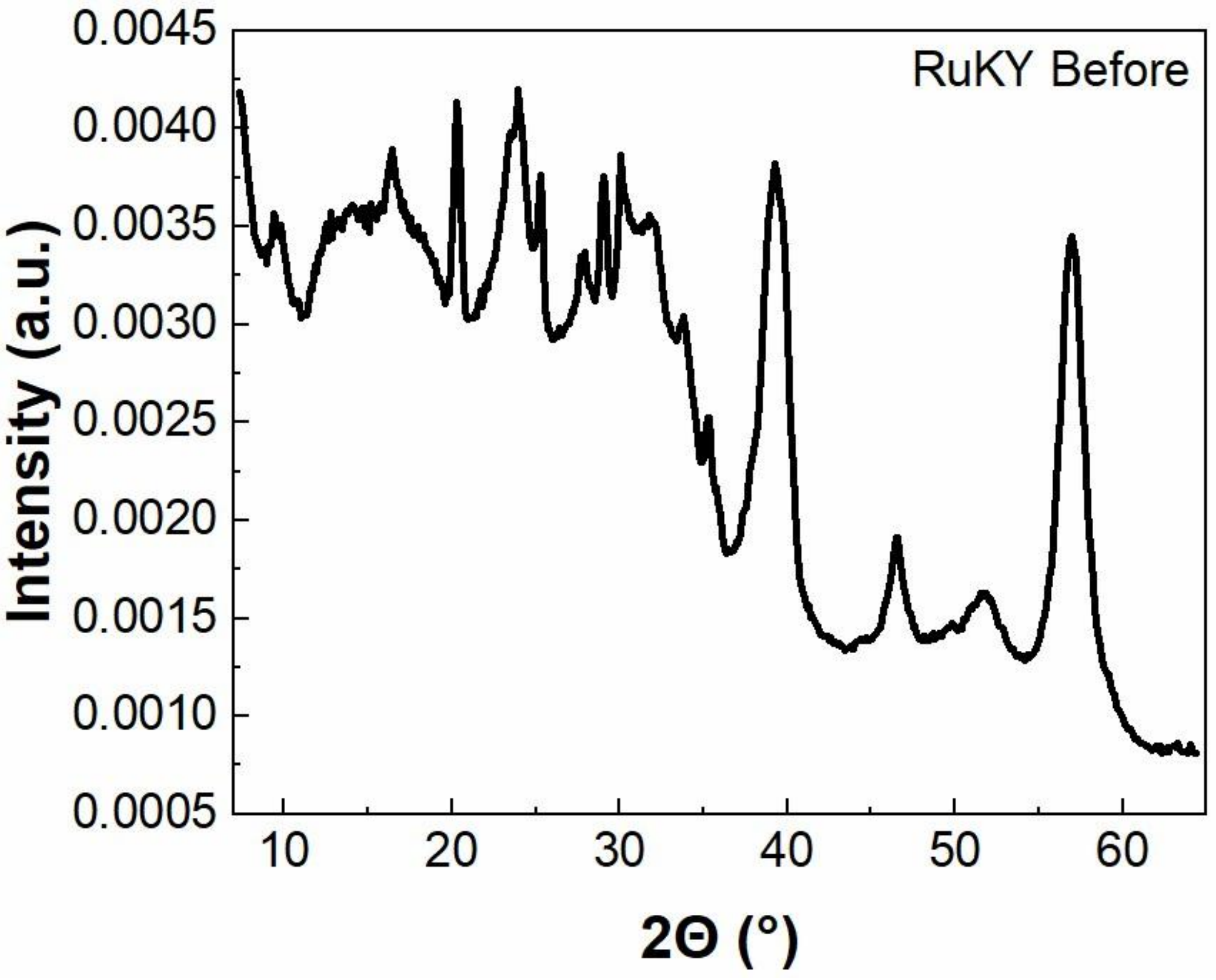

Figure S3. WAXS spectrum (where the x-axis is converted to 2Θ with a copper laser source) of the 3%Ru/1%Y/12%K/γ-Al_2O_3 catalyst before reaction.

Table S1. Phase and d spacing data corresponding to Figure S3.

2Θ(°)	d-spacing (Å)	Phase (h,k,l)
9.48	9.32	-
16.46	5.38	$K_{0.33}Al_3O_{4.67}$ (1,1,0)
20.30	4.37	Y_2O_3 (2,1,1)
23.48	3.79	$K_{0.33}Al_3O_{4.67}$ (0,2,0)
23.94	3.71	$K_{0.33}Al_3O_{4.67}$ (2,0,0)
25.27	3.52	$K_{0.33}Al_3O_{4.67}$ (2,1,0)
27.78	3.21	RuO_2 (1,1,0)
28.96	3.08	Y_2O_3 (2,2,2)
30.25	2.95	$K_{0.33}Al_3O_{4.67}$ (0,1,1)
31.80	2.81	γ-Al_2O_3 (2,2,0)
33.74	2.65	Y_2O_3 (4,0,0)
35.38	2.54	RuO_2 (1,1,0)
39.27	2.23	γ-Al_2O_3 (2,2,2)<br>RuO_2 (1,1,1)
46.53	1.95	γ-Al_2O_3 (4,0,0)
51.61	1.77	γ-Al_2O_3 (3,3,1)
56.93	1.62	γ-Al_2O_3 (4,2,2)

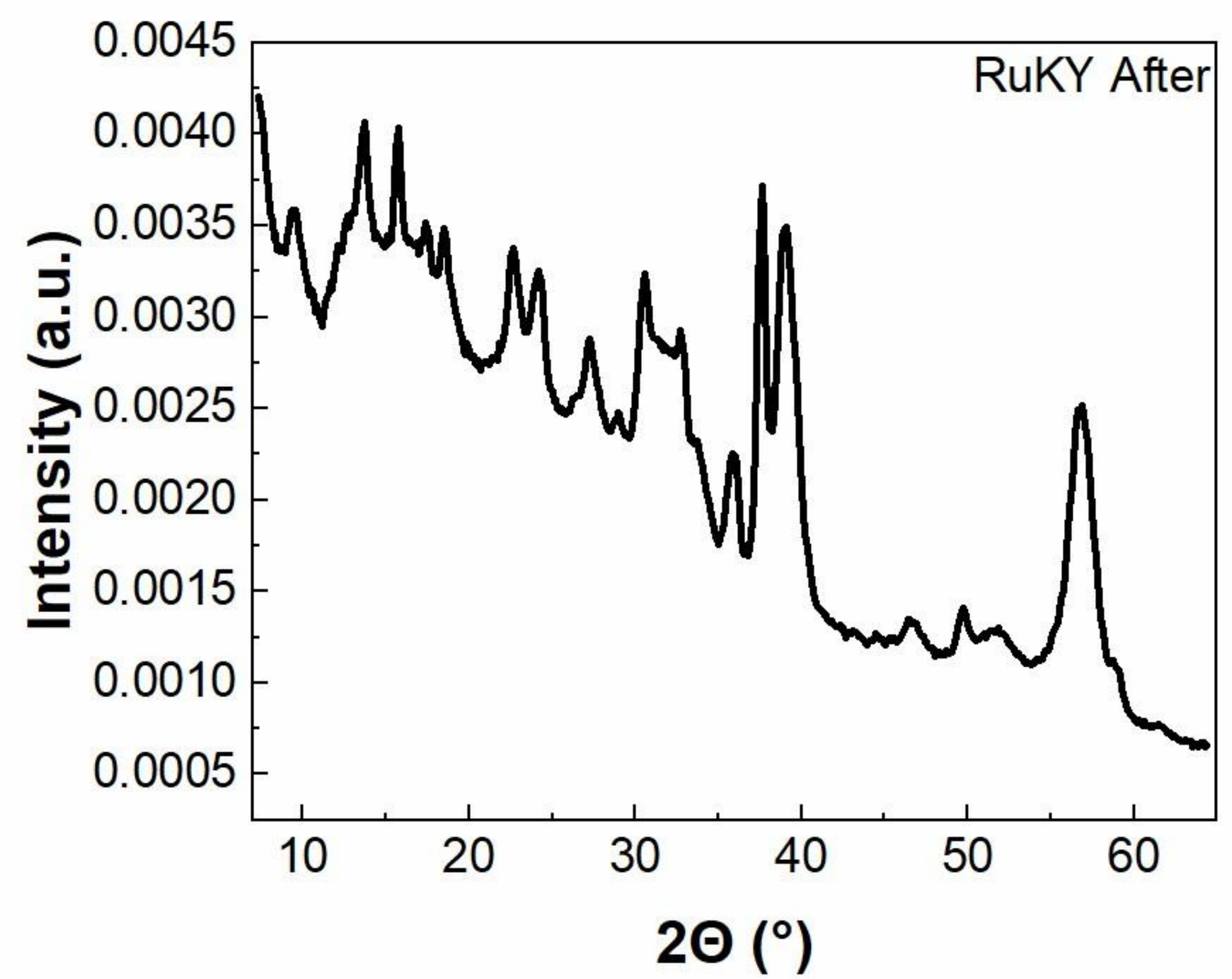

Figure S4. WAXS spectrum (where the x-axis is converted to 2Θ with a copper laser source) of the 3%Ru/1%Y/12%K/γ-Al_2O_3 catalyst after reaction.

Table S2. Phase and d spacing data corresponding to Figure S4.

2Θ(°)	d-spacing (Å)	Phase (h,k,l)
9.42	9.38	-
13.64	6.49	-
15.71	5.64	$K_{1.15}Al_{10.95}O_{17}$ (0,0,4)
17.36	5.11	$K_{0.33}Al_3O_{4.67}$ (1,1,0)
18.49	4.79	$K_{1.15}Al_{10.95}O_{17}$ (1,0,1)
22.68	3.92	$K_{1.15}Al_{10.95}O_{17}$ (1,0,3)
24.14	3.68	Y_2O_3 (2,2,0)
27.25	3.27	RuO_2 (1,1,0)
28.98	3.07	Y_2O_3 (2,2,2)
30.54	2.93	$K_{0.33}Al_3O_{4.67}$ (0,1,1)
32.76	2.73	$K_{1.15}Al_{10.95}O_{17}$ (1,1,2)
33.25	2.69	$K_{1.15}Al_{10.95}O_{17}$ (1,0,7)
35.88	2.50	Y_2O_3 (4,1,1)
37.60	2.39	γ-Al_2O_3 (3,1,1)
39.07	2.30	γ-Al_2O_3 (2,2,2)
46.47	1.95	$K_{1.15}Al_{10.95}O_{17}$ (2,0,7)
49.73	1.83	γ-Al_2O_3 (3,3,1)
51.79	1.76	$K_{1.15}Al_{10.95}O_{17}$ (1,1,10)
56.83	1.62	γ-Al_2O_3 (4,2,2)
58.86	1.57	Ru (1,0,2)

Kinetic Isotope Effect

The following figures (Figure S5-19) are the various conditions testing that was used to construct the Arrhenius plot and rate plots with NH_3 as the reagents. Tables of turnover frequencies and hydrogen productivities are provided and a table corresponding to 450°C is shown in Table SX, while the figure is in the main text in Figure 1E. The figures (Figure S20-33) all utilize ND_3 as the reagent and have subsequent tables with turnover frequency and deuterium production. Turnover frequency was calculated with the total amount of metal on the catalyst not just the active metal, ruthenium.

Table S3. The turnover frequency and hydrogen productivity of the RuKY catalyst at 450°C.

Ammonia GHSV ($mL_{NH3}/hr/g_{cat}$)	Turnover Frequency (s^{-1})	Hydrogen Productivity ($mol_{H2}/g_{cat}/hr$)
2400	0.43	0.134
3600	0.61	0.190
4800	0.76	0.238
6000	0.90	0.281
7200	1.01	0.318
8400	1.12	0.352
9600	1.23	0.384
10800	1.32	0.412

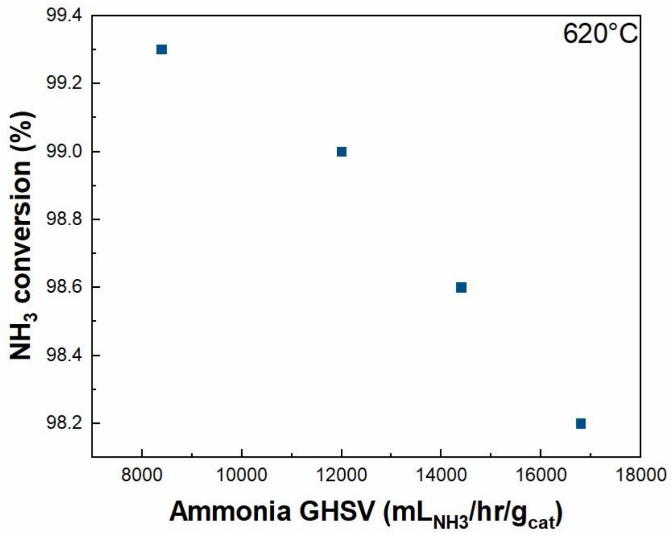

Figure S5. The conversion of NH_3 at 620°C at various pure ammonia flow rates that were used to construct the rate plot.

Table S4. The turnover frequency and hydrogen productivity of the RuKY catalyst at 620°C.

Ammonia GHSV	Turnover Frequency (s^{-1})	Hydrogen Productivity

$(mL_{NH3}/hr/g_{cat})$		$(mol_{H2}/g_{cat}/hr)$
8400	1.78	0.559
12000	2.54	0.795
14400	3.04	0.951
16800	3.53	1.105

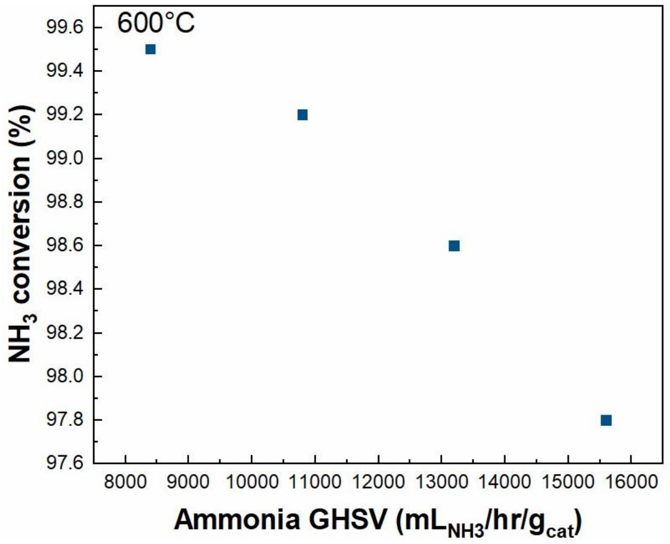

Figure S6. The conversion of NH_3 at 600°C at various pure ammonia flow rates that were used to construct the rate plot.

Table S5. The turnover frequency and hydrogen productivity of the RuKY catalyst at 600°C.

Ammonia GHSV $(mL_{NH3}/hr/g_{cat})$	Turnover Frequency (s^{-1})	Hydrogen Productivity $(mol_{H2}/g_{cat}/hr)$
8400	1.79	0.560
10800	2.29	0.717
13200	2.78	0.872
15600	3.26	1.021

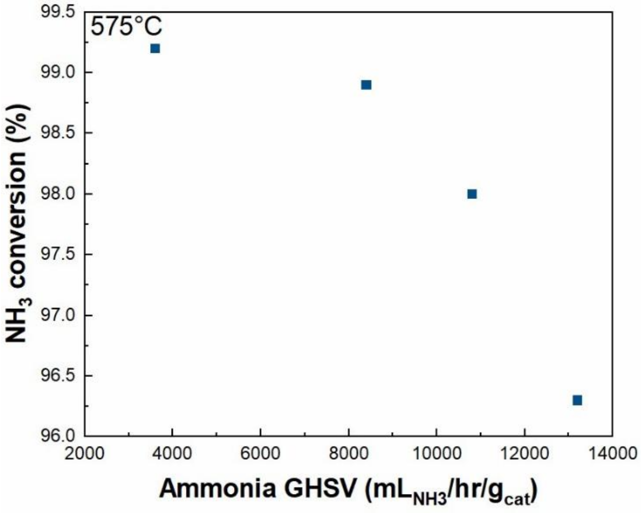

Figure S7. The conversion of NH_3 at 575°C at various pure ammonia flow rates that were used to construct the rate plot.

Table S6. The turnover frequency and hydrogen productivity of the RuKY catalyst at 575°C.

Ammonia GHSV ($mL_{NH3}/hr/g_{cat}$)	Turnover Frequency (s^{-1})	Hydrogen Productivity ($mol_{H2}/g_{cat}/hr$)
3600	0.76	0.239
8400	1.78	0.556
10800	2.26	0.709
13200	2.72	0.851

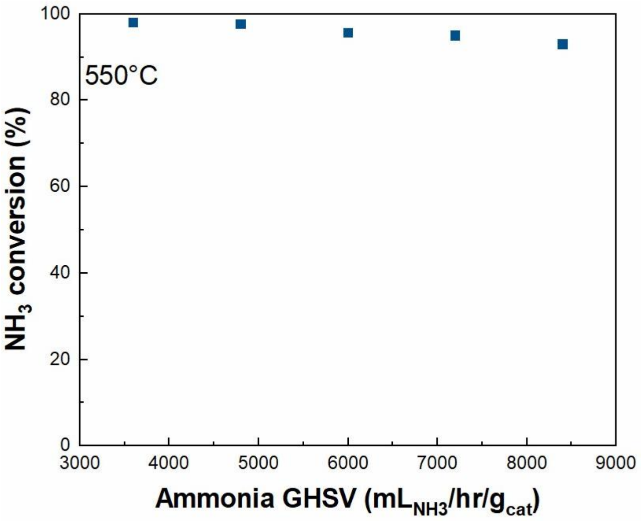

Figure S8. The conversion of NH_3 at 550°C at various pure ammonia flow rates that were used to construct the rate plot.

Table S7. The turnover frequency and hydrogen productivity of the RuKY catalyst at 550°C.

Ammonia GHSV ($mL_{NH3}/hr/g_{cat}$)	Turnover Frequency (s^{-1})	Hydrogen Productivity ($mol_{H2}/g_{cat}/hr$)
3600	0.75	0.236
4800	1.00	0.314
6000	1.24	0.388
7200	1.46	0.458
8400	1.67	0.523

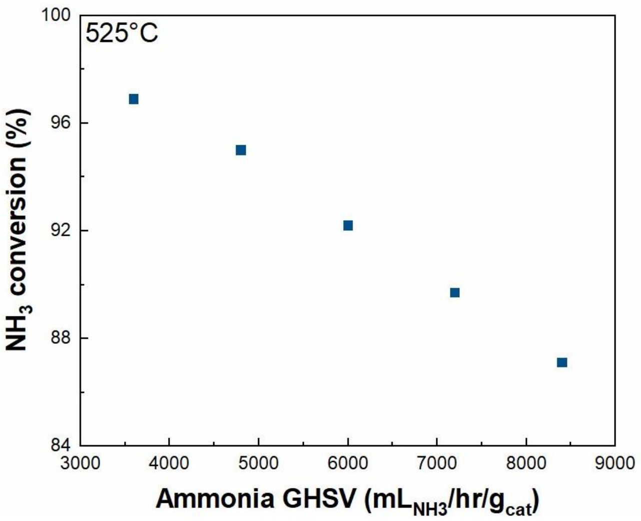

Figure S9. The conversion of NH_3 at 525°C at various pure ammonia flow rates that were used to construct the rate plot.

Table S8. The turnover frequency and hydrogen productivity of the RuKY catalyst at 525°C.

Ammonia GHSV ($mL_{NH3}/hr/g_{cat}$)	Turnover Frequency (s^{-1})	Hydrogen Productivity ($mol_{H2}/g_{cat}/hr$)
3600	0.75	0.234
4800	0.98	0.305
6000	1.18	0.370
7200	1.38	0.432
8400	1.56	0.490

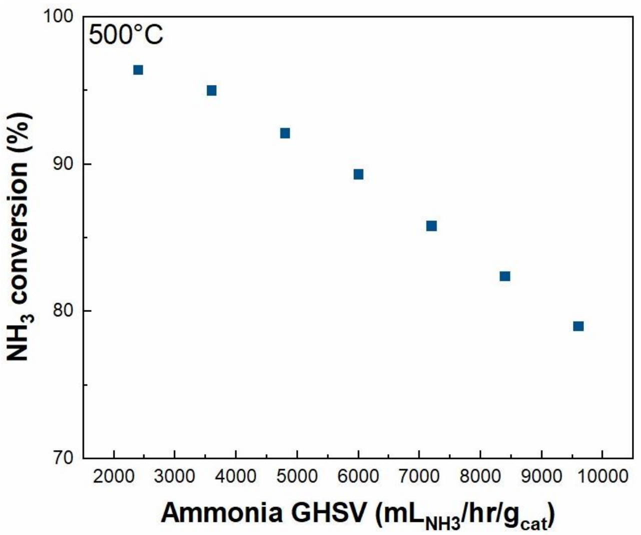

Figure S10. The conversion of NH_3 at 500°C at various pure ammonia flow rates that were used to construct the rate plot.

Table S9. The turnover frequency and hydrogen productivity of the RuKY catalyst at 500°C.

Ammonia GHSV ($mL_{NH3}/hr/g_{cat}$)	Turnover Frequency (s^{-1})	Hydrogen Productivity ($mol_{H2}/g_{cat}/hr$)
2400	0.49	0.155
3600	0.73	0.229
4800	0.95	0.296
6000	1.15	0.359
7200	1.32	0.414
8400	1.48	0.464
9600	1.62	0.508

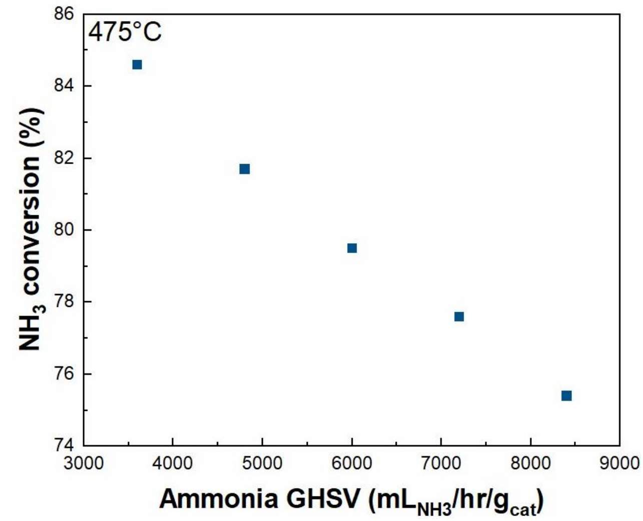

Figure S11. The conversion of NH_3 at 475°C at various pure ammonia flow rates that were used to construct the rate plot.

Table S10. The turnover frequency and hydrogen productivity of the RuKY catalyst at 475°C.

Ammonia GHSV ($mL_{NH3}/hr/g_{cat}$)	Turnover Frequency (s^{-1})	Hydrogen Productivity ($mol_{H2}/g_{cat}/hr$)
3600	0.65	0.203
4800	0.84	0.263
6000	1.02	0.320
7200	1.19	0.373
8400	1.36	0.424

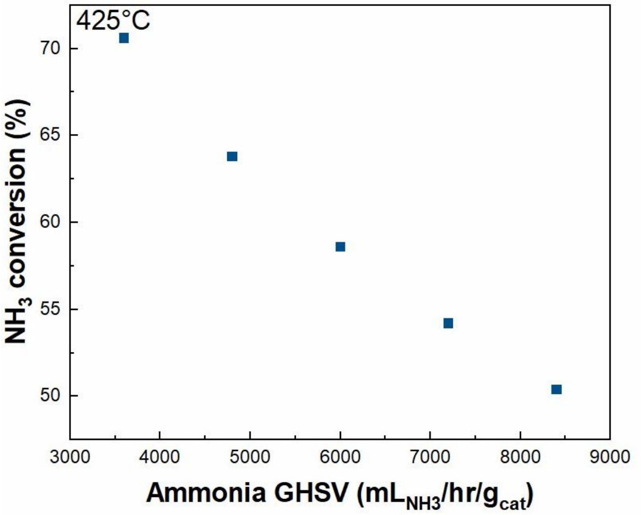

Figure S12. The conversion of NH_3 at 425°C at various pure ammonia flow rates that were used to construct the rate plot.

Table S11. The turnover frequency and hydrogen productivity of the RuKY catalyst at 425°C.

Ammonia GHSV (mL_{NH3}/hr/g_{cat})	Turnover Frequency (s^{-1})	Hydrogen Productivity (mol_{H2}/g_{cat}/hr)
3600	0.54	0.170
4800	0.66	0.205
6000	0.75	0.235
7200	0.83	0.261
8400	0.91	0.284

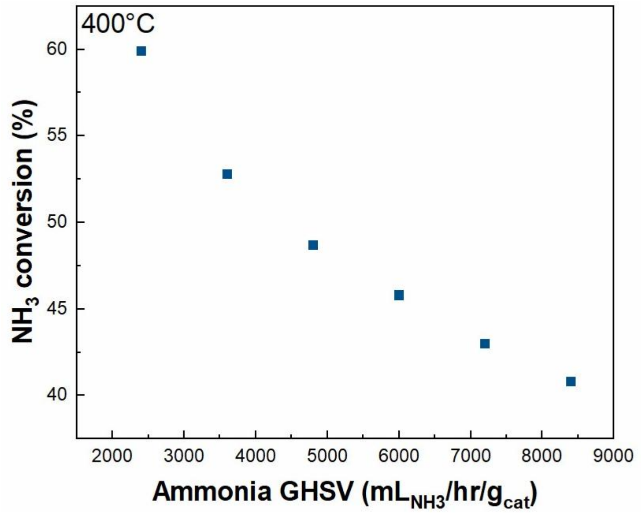

Figure S13. The conversion of NH_3 at 400°C at various pure ammonia flow rates that were used to construct the rate plot.

Table S12. The turnover frequency and hydrogen productivity of the RuKY catalyst at 400°C.

Ammonia GHSV (mL_{NH3}/hr/g_{cat})	Turnover Frequency (s^{-1})	Hydrogen Productivity (mol_{H2}/g_{cat}/hr)
2400	0.31	0.096
3600	0.41	0.127
4800	0.50	0.157
6000	0.59	0.184
7200	0.66	0.207
8400	0.73	0.230

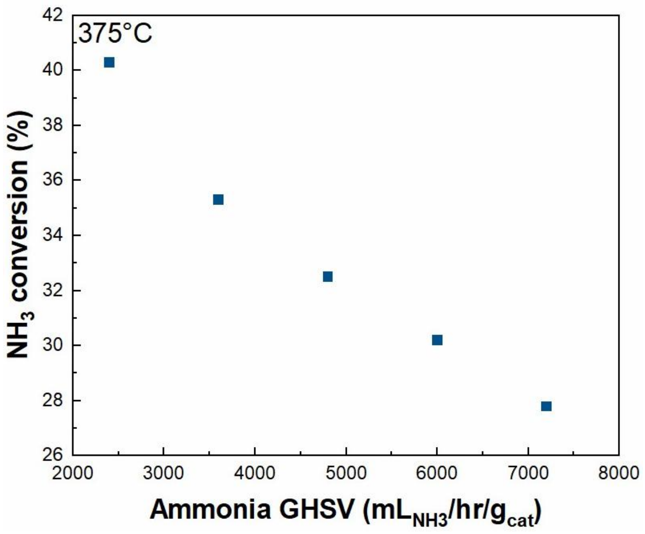

Figure S14. The conversion of NH_3 at 375°C at various pure ammonia flow rates that were used to construct the rate plot.

Table S13. The turnover frequency and hydrogen productivity of the RuKY catalyst at 375°C.

Ammonia GHSV ($mL_{NH3}/hr/g_{cat}$)	Turnover Frequency (s^{-1})	Hydrogen Productivity ($mol_{H2}/g_{cat}/hr$)
2400	0.21	0.065
3600	0.27	0.085
4800	0.33	0.105
6000	0.39	0.121
7200	0.43	0.134

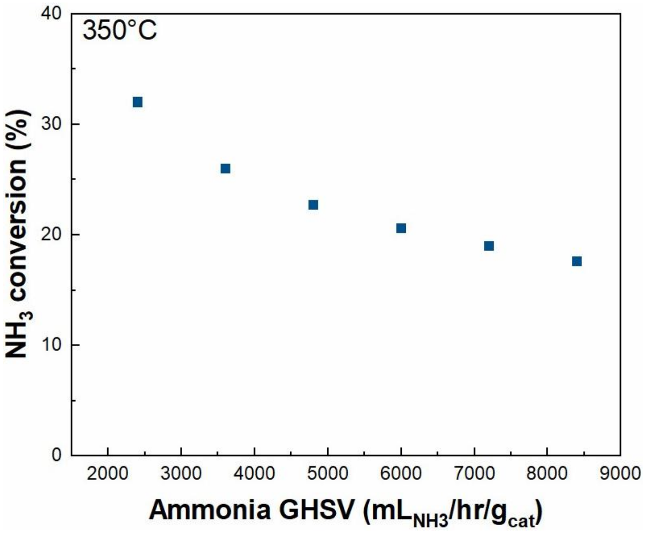

Figure S15. The conversion of NH_3 at 350°C at various pure ammonia flow rates that were used to construct the rate plot.

Table S14. The turnover frequency and hydrogen productivity of the RuKY catalyst at 350°C.

Ammonia GHSV ($mL_{NH3}/hr/g_{cat}$)	Turnover Frequency (s^{-1})	Hydrogen Productivity ($mol_{H2}/g_{cat}/hr$)
2400	0.16	0.051
3600	0.20	0.063
4800	0.23	0.073
6000	0.26	0.083
7200	0.29	0.091
8400	0.32	0.099

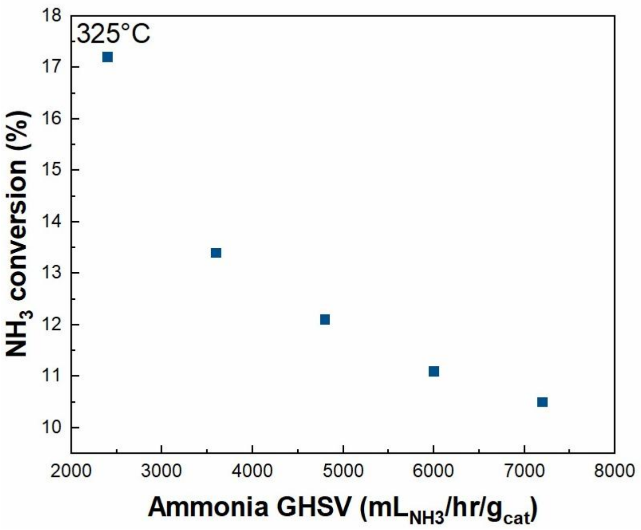

Figure S16. The conversion of NH_3 at 325°C at various pure ammonia flow rates that were used to construct the rate plot.

Table S15. The turnover frequency and hydrogen productivity of the RuKY catalyst at 325°C.

Ammonia GHSV ($mL_{NH3}/hr/g_{cat}$)	Turnover Frequency (s^{-1})	Hydrogen Productivity ($mol_{H2}/g_{cat}/hr$)
2400	0.09	0.028
3600	0.10	0.032
4800	0.12	0.039
6000	0.14	0.045
7200	0.16	0.051

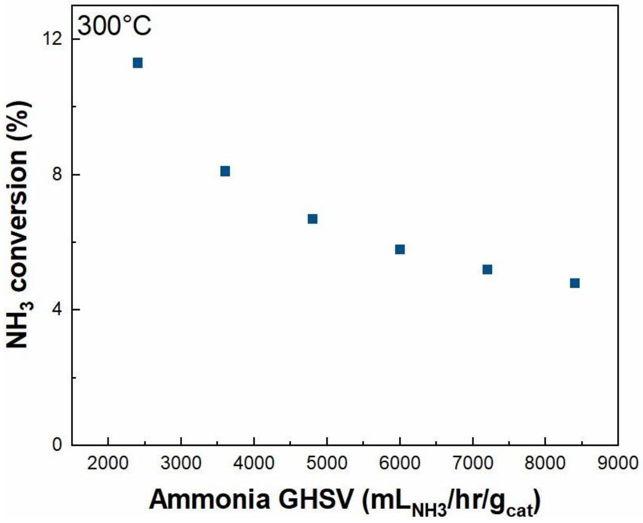

Figure S17. The conversion of NH_3 at 300°C at various pure ammonia flow rates that were used to construct the rate plot.

Table S16. The turnover frequency and hydrogen productivity of the RuKY catalyst at 300°C.

Ammonia GHSV ($mL_{NH3}/hr/g_{cat}$)	Turnover Frequency (s^{-1})	Hydrogen Productivity ($mol_{H2}/g_{cat}/hr$)
2400	0.06	0.018
3600	0.06	0.020
4800	0.07	0.022
6000	0.07	0.023
7200	0.08	0.025
8400	0.09	0.027

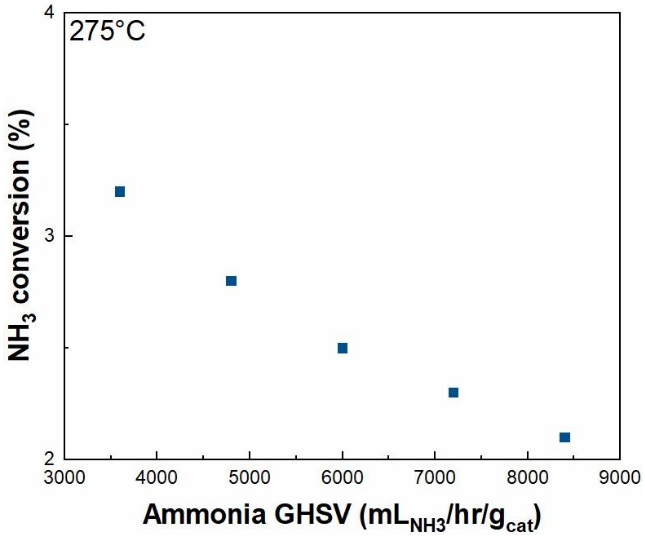

Figure S18. The conversion of NH_3 at 275°C at various pure ammonia flow rates that were used to construct the rate plot.

Table S17. The turnover frequency and hydrogen productivity of the RuKY catalyst at 275°C.

Ammonia GHSV ($mL_{NH3}/hr/g_{cat}$)	Turnover Frequency (s^{-1})	Hydrogen Productivity ($mol_{H2}/g_{cat}/hr$)
3600	0.02	0.008
4800	0.03	0.009
6000	0.03	0.010
7200	0.04	0.011
8400	0.04	0.012

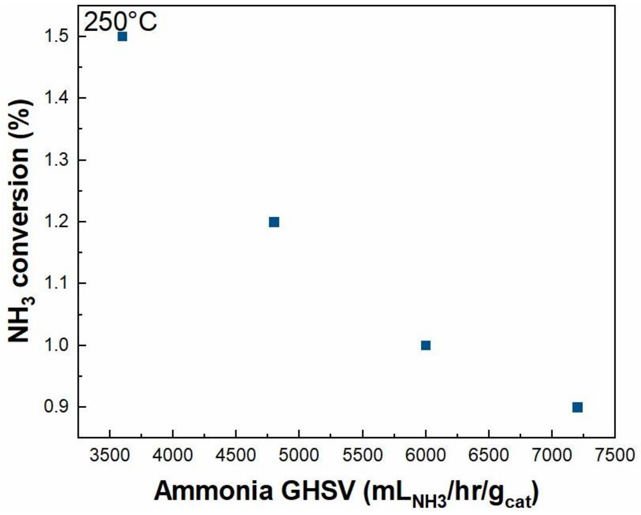

Figure S19. The conversion of NH_3 at 250°C at various pure ammonia flow rates that were used to construct the rate plot.

Table S18. The turnover frequency and hydrogen productivity of the RuKY catalyst at 250°C.

Ammonia GHSV ($mL_{NH3}/hr/g_{cat}$)	Turnover Frequency (s^{-1})	Hydrogen Productivity ($mol_{H2}/g_{cat}/hr$)
3600	0.01	0.004
4800	0.01	0.004
6000	0.01	0.004
7200	0.01	0.004

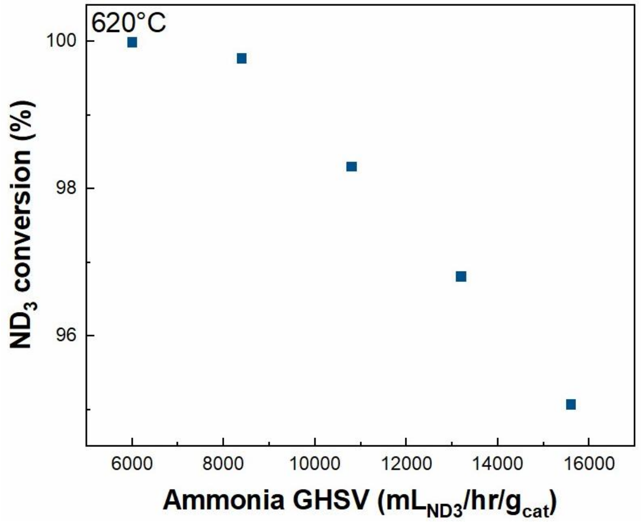

Figure S20. The conversion of ND_3 at 620°C at various pure ammonia flow rates that were used to construct the rate plot.

Table S19. The turnover frequency and deuterium productivity of the RuKY catalyst at 620°C.

Ammonia GHSV (mL_{NH3}/hr/g_{cat})	Turnover Frequency (s^{-1})	Deuterium Productivity (mol_{D2}/g_{cat}/hr)
6000	1.28	0.402
8400	1.79	0.561
10800	2.27	0.711
13200	2.73	0.856
15600	3.17	0.993

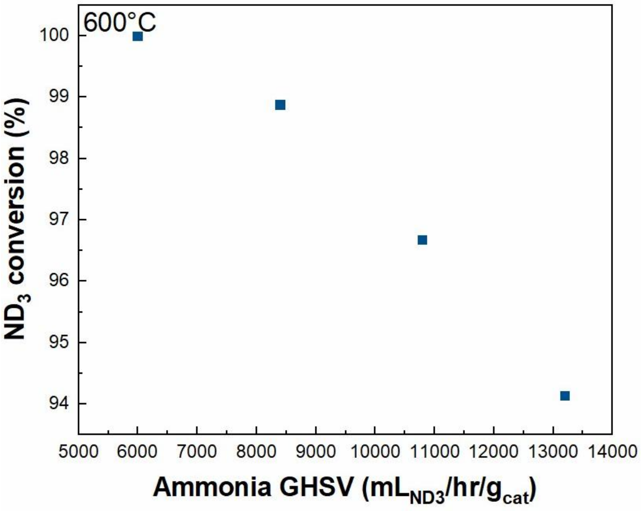

Figure S21. The conversion of ND_3 at 600°C at various pure ammonia flow rates that were used to construct the rate plot.

Table S20. The turnover frequency and deterium productivity of the RuKY catalyst at 600°C.

Ammonia GHSV (mL_{NH3}/hr/g_{cat})	Turnover Frequency (s^{-1})	Deuterium Productivity (mol_{D2}/g_{cat}/hr)
6000	1.28	0.402
8400	1.78	0.556
10800	2.24	0.700
13200	2.66	0.832

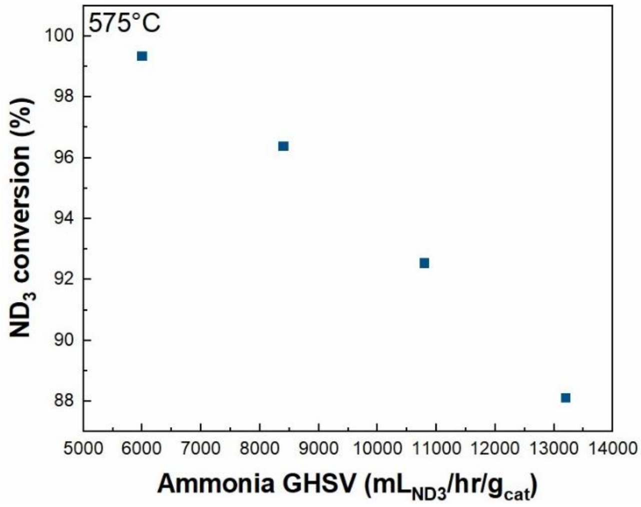

Figure S22. The conversion of ND_3 at 575°C at various pure ammonia flow rates that were used to construct the rate plot.

Table S21. The turnover frequency and deuterium productivity of the RuKY catalyst at 575°C.

Ammonia GHSV (mL_{NH3}/hr/g_{cat})	Turnover Frequency (s^{-1})	Deuterium Productivity (mol_{D2}/g_{cat}/hr)
6000	1.27	0.399
8400	0.73	0.542
10800	2.14	0.669
13200	2.49	0.779

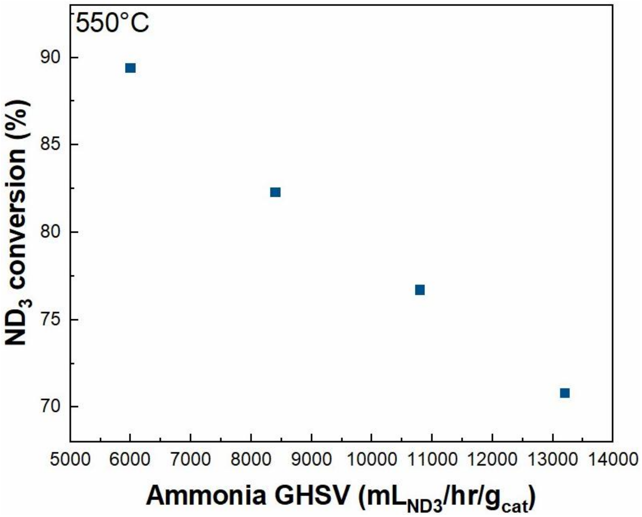

Figure S23. The conversion of ND_3 at 550°C at various pure ammonia flow rates that were used to construct the rate plot.

Table S22. The turnover frequency and deuterium productivity of the RuKY catalyst at 550°C.

Ammonia GHSV ($mL_{NH3}/hr/g_{cat}$)	Turnover Frequency (s^{-1})	Deuterium Productivity ($mol_{D2}/g_{cat}/hr$)
6000	1.15	0.359
8400	1.49	0.466
10800	1.77	0.554
13200	2.00	0.626

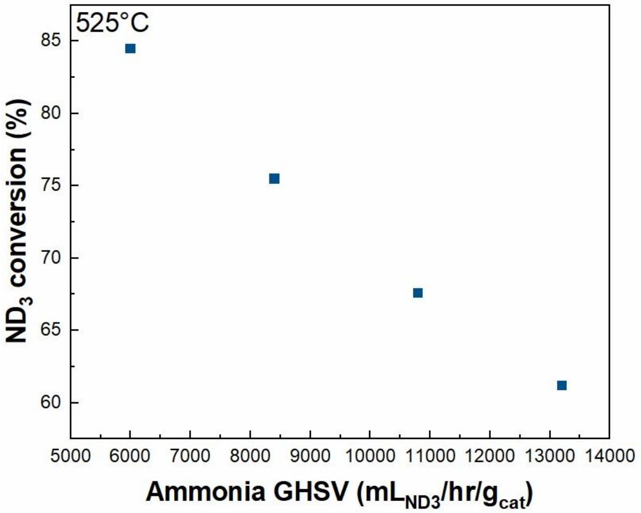

Figure S24. The conversion of ND_3 at 525°C at various pure ammonia flow rates that were used to construct the rate plot.

Table S23. The turnover frequency and deuterium productivity of the RuKY catalyst at 525°C.

Ammonia GHSV ($mL_{NH3}/hr/g_{cat}$)	Turnover Frequency (s^{-1})	Deuterium Productivity ($mol_{D2}/g_{cat}/hr$)
6000	1.08	0.340
8400	1.36	0.425
10800	1.56	0.489
13200	1.73	0.541

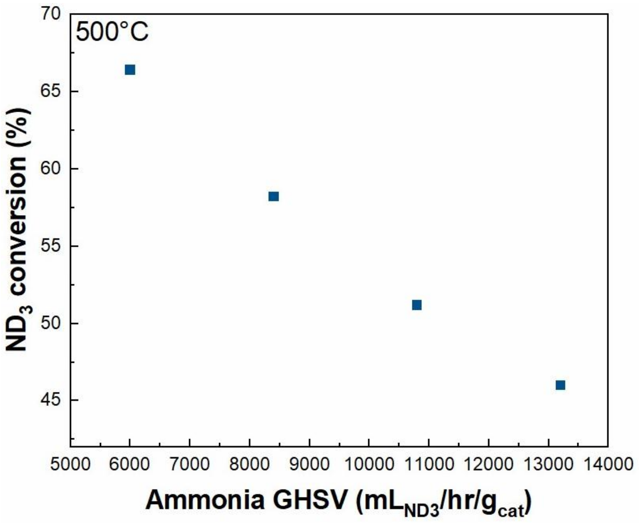

Figure S25. The conversion of ND_3 at 500°C at various pure ammonia flow rates that were used to construct the rate plot.

Table S24. The turnover frequency and deuterium productivity of the RuKY catalyst at 500°C.

Ammonia GHSV ($mL_{NH3}/hr/g_{cat}$)	Turnover Frequency (s^{-1})	Deuterium Productivity ($mol_{D2}/g_{cat}/hr$)
6000	0.85	0.267
8400	1.05	0.327
10800	1.18	0.370
13200	1.30	0.406

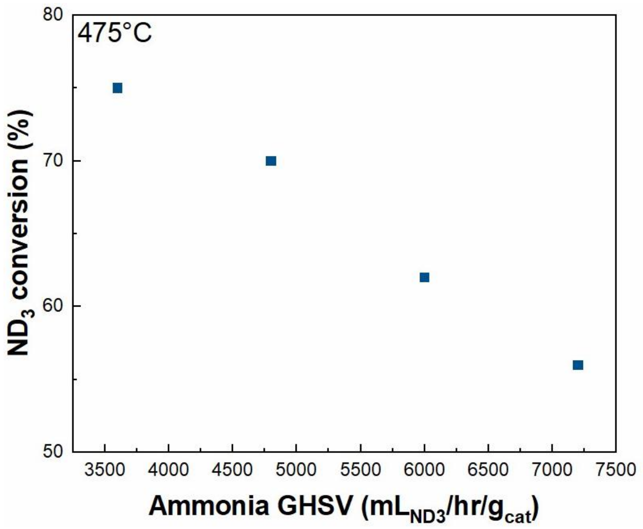

Figure S26. The conversion of ND_3 at 475°C at various pure ammonia flow rates that were used to construct the rate plot.

Table S25. The turnover frequency and deuterium productivity of the RuKY catalyst at 475°C.

Ammonia GHSV ($mL_{NH3}/hr/g_{cat}$)	Turnover Frequency (s^{-1})	Deuterium Productivity ($mol_{D2}/g_{cat}/hr$)
3600	0.58	0.181
4800	0.72	0.225
6000	0.80	0.249
7200	0.86	0.270

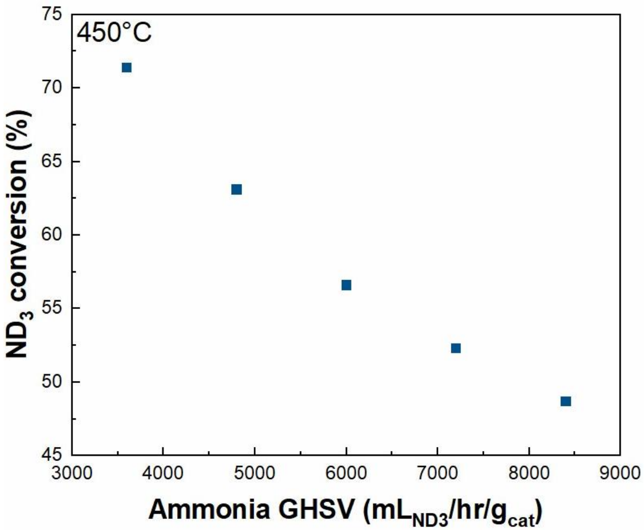

Figure S27. The conversion of ND_3 at 450°C at various pure ammonia flow rates that were used to construct the rate plot.

Table S26. The turnover frequency and deuterium productivity of the RuKY catalyst at 450°C.

Ammonia GHSV ($mL_{NH3}/hr/g_{cat}$)	Turnover Frequency (s^{-1})	Deuterium Productivity ($mol_{D2}/g_{cat}/hr$)
3600	0.55	0.172
4800	0.65	0.203
6000	0.73	0.228
7200	0.81	0.252
8400	0.87	0.274

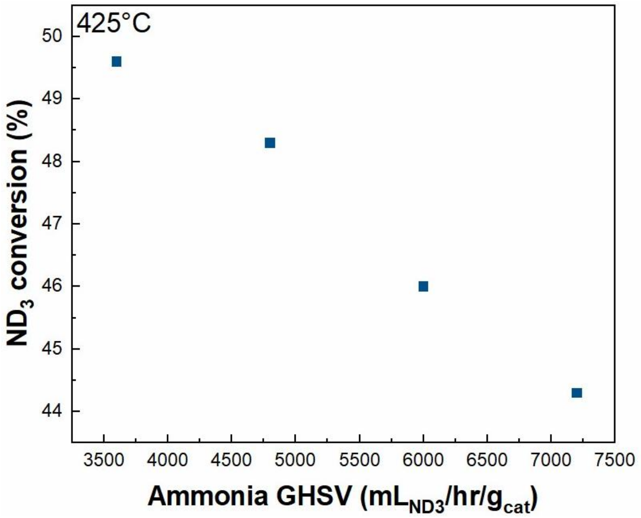

Figure S28. The conversion of ND_3 at 425°C at various pure ammonia flow rates that were used to construct the rate plot.

Table S27. The turnover frequency and deuterium productivity of the RuKY catalyst at 425°C.

Ammonia GHSV ($mL_{NH3}/hr/g_{cat}$)	Turnover Frequency (s^{-1})	Deuterium Productivity ($mol_{D2}/g_{cat}/hr$)
6000	0.38	0.119
8400	0.50	0.155
10800	0.59	0.185
13200	0.68	0.214

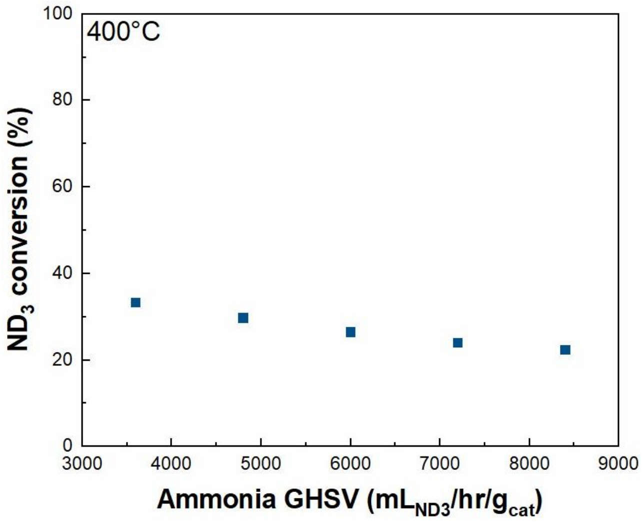

Figure S29. The conversion of ND_3 at 400°C at various pure ammonia flow rates that were used to construct the rate plot.

Table S28. The turnover frequency and deuterium productivity of the RuKY catalyst at 400°C.

Ammonia GHSV ($mL_{NH3}/hr/g_{cat}$)	Turnover Frequency (s^{-1})	Deuterium Productivity ($mol_{D2}/g_{cat}/hr$)
2400	0.26	0.080
3600	0.31	0.096
4800	0.34	0.106
6000	0.37	0.116
7200	0.40	0.126

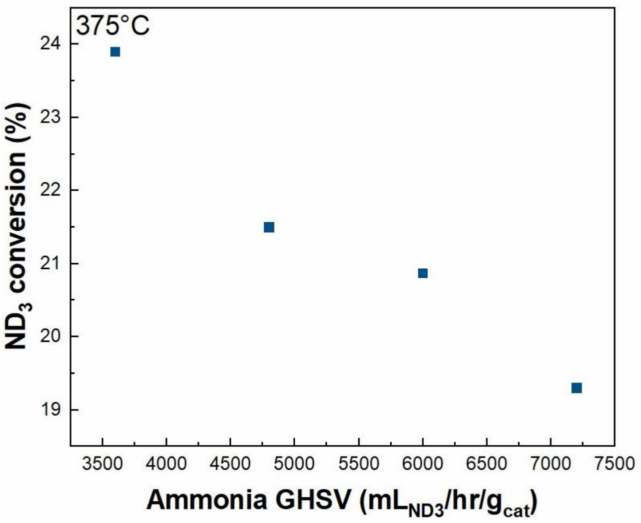

Figure S30. The conversion of ND_3 at 375°C at various pure ammonia flow rates that were used to construct the rate plot.

Table S29. The turnover frequency and deuterium productivity of the RuKY catalyst at 375°C.

Ammonia GHSV ($mL_{NH3}/hr/g_{cat}$)	Turnover Frequency (s^{-1})	Deuterium Productivity ($mol_{D2}/g_{cat}/hr$)
3600	0.18	0.058
4800	0.22	0.069
6000	0.27	0.084
7200	0.30	0.093

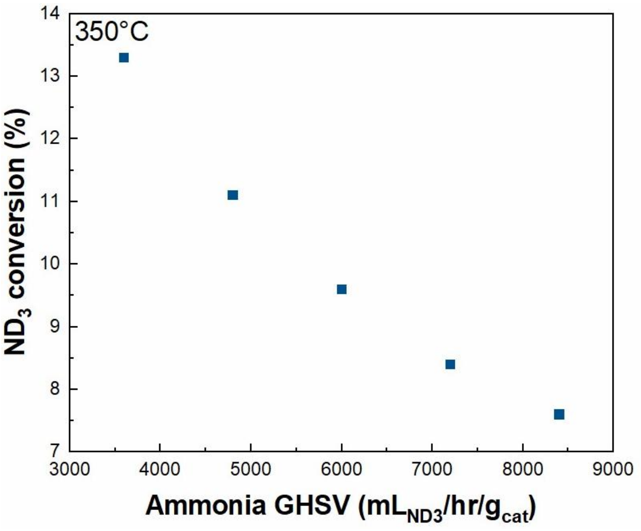

Figure S31. The conversion of ND_3 at 350°C at various pure ammonia flow rates that were used to construct the rate plot.

Table S30. The turnover frequency and deuterium productivity of the RuKY catalyst at 350°C.

Ammonia GHSV ($mL_{NH3}/hr/g_{cat}$)	Turnover Frequency (s^{-1})	Deuterium Productivity ($mol_{D2}/g_{cat}/hr$)
2400	0.10	0.032
3600	0.11	0.036
4800	0.12	0.038
6000	0.13	0.041
7200	0.14	0.043

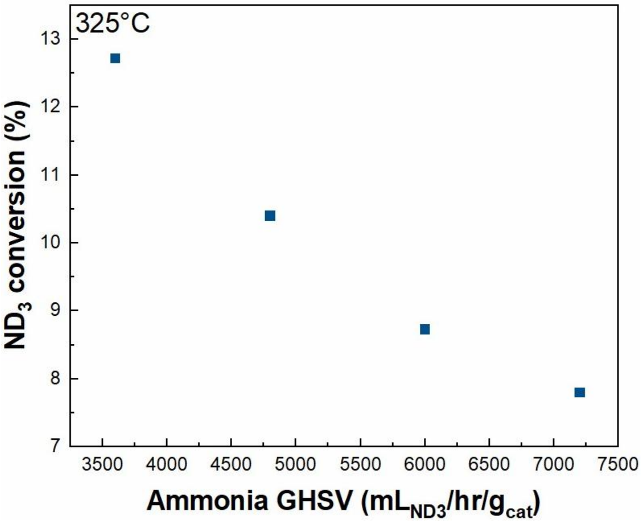

Figure S32. The conversion of ND_3 at 325°C at various pure ammonia flow rates that were used to construct the rate plot.

Table S31. The turnover frequency and deuterium productivity of the RuKY catalyst at 325°C.

Ammonia GHSV ($mL_{NH3}/hr/g_{cat}$)	Turnover Frequency (s^{-1})	Deuterium Productivity ($mol_{D2}/g_{cat}/hr$)
3600	0.10	0.031
4800	0.11	0.033
6000	0.11	0.035
7200	0.12	0.038

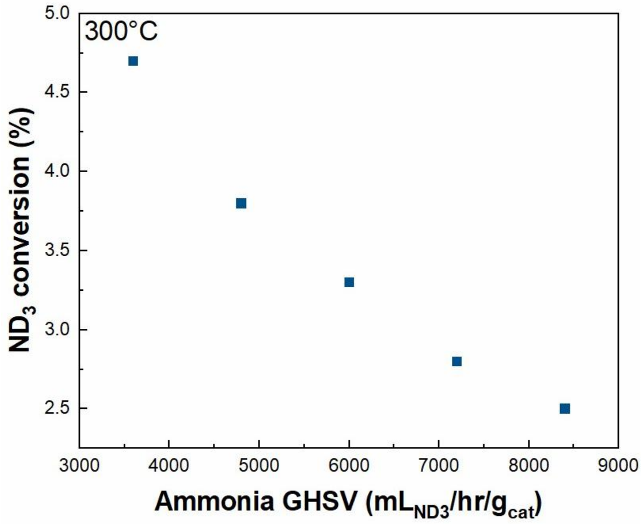

Figure S33. The conversion of ND_3 at 300°C at various pure ammonia flow rates that were used to construct the rate plot.

Table S32. The turnover frequency and deuterium productivity of the RuKY catalyst at 300°C.

Ammonia GHSV ($mL_{NH3}/hr/g_{cat}$)	Turnover Frequency (s^{-1})	Deuterium Productivity ($mol_{D2}/g_{cat}/hr$)
3600	0.04	0.011
4800	0.04	0.012
6000	0.04	0.013
7200	0.04	0.014
8400	0.04	0.014

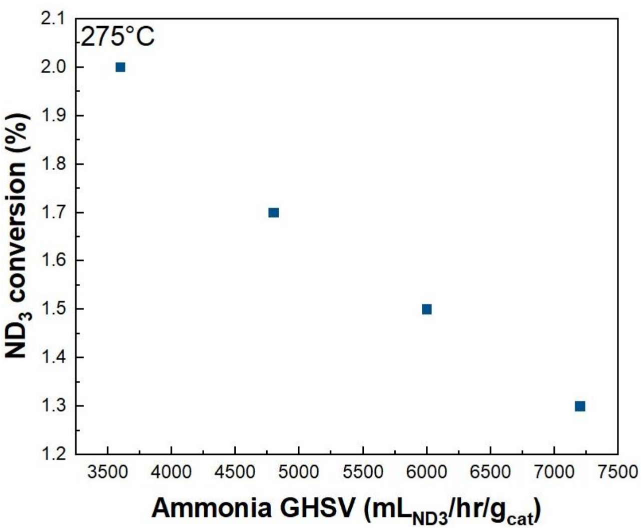

Figure S34. The conversion of ND_3 at 275°C at various pure ammonia flow rates that were used to construct the rate plot.

Table S33. The turnover frequency and deuterium productivity of the RuKY catalyst at 275°C.

Ammonia GHSV ($mL_{NH3}/hr/g_{cat}$)	Turnover Frequency (s^{-1})	Deuterium Productivity ($mol_{D2}/g_{cat}/hr$)
3600	0.02	0.005
4800	0.02	0.005
6000	0.02	0.005
7200	0.02	0.006

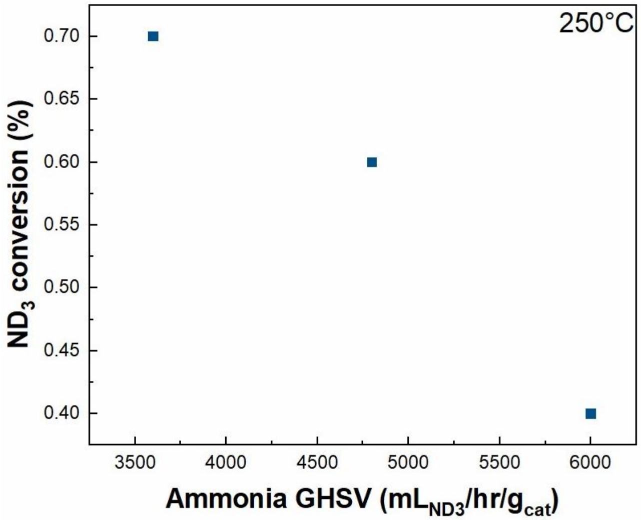

Figure S35. The conversion of ND_3 at 250°C at various pure ammonia flow rates that were used to construct the rate plot.

Table S34. The turnover frequency and deuterium productivity of the RuKY catalyst at 250°C.

Ammonia GHSV ($mL_{NH3}/hr/g_{cat}$)	Turnover Frequency (s^{-1})	Deuterium Productivity ($mol_{D2}/g_{cat}/hr$)
3600	0.01	0.002
4800	0.01	0.002
6000	0.01	0.002

Table S35 and

Table S*36* contain the rate constants and KIE for all of the temperatures tested. At each temperature NH_3 performed better than ND_3.

Table S35. Second order rate constants for the RuKY catalyst for both NH_3 and ND_3 as the reactant.

Temperature (°C)	k_H *(L/mol·s)*	k_D *(L/mol·s)*	*KIE* (k_H/k_D)
250	2.98	1.31	2.275
275	10.20	6.02	1.694
300	20.91	11.01	1.899
325	42.65	38.16	1.118
350	127.2	43.43	2.929
375	237.9	123.8	1.922
400	742.7	225.3	3.296
425	1992	795.2	2.505
450	4104	2035	2.017
475	7681	5357	1.434

Table S36. First order rate constants for the RuKY catalyst for both NH_3 and ND_3 as the reactant.

Temperature (°C)	k_H (s^{-1})	k_D (s^{-1})	*KIE* (k_H/k_D)
500	33.08	14.33	2.308
525	43.38	27.37	1.585
550	61.03	35.97	1.697
575	117.3	78.14	1.501
600	163.0	111.2	1.466
620	176.4	146.3	1.206

Additional Permeation Membrane Data

To test the effect of hydrogen on the permeation membrane reactor (as shown in Figure S36), the feed gas was varied to include increasing amount of hydrogen. At each temperature tested, the amount of hydrogen added had an adverse effect on the ammonia cracking, indicating that the PMR is limited by the hydrogen permeation ability of the membrane rather than the catalyst and its performance.

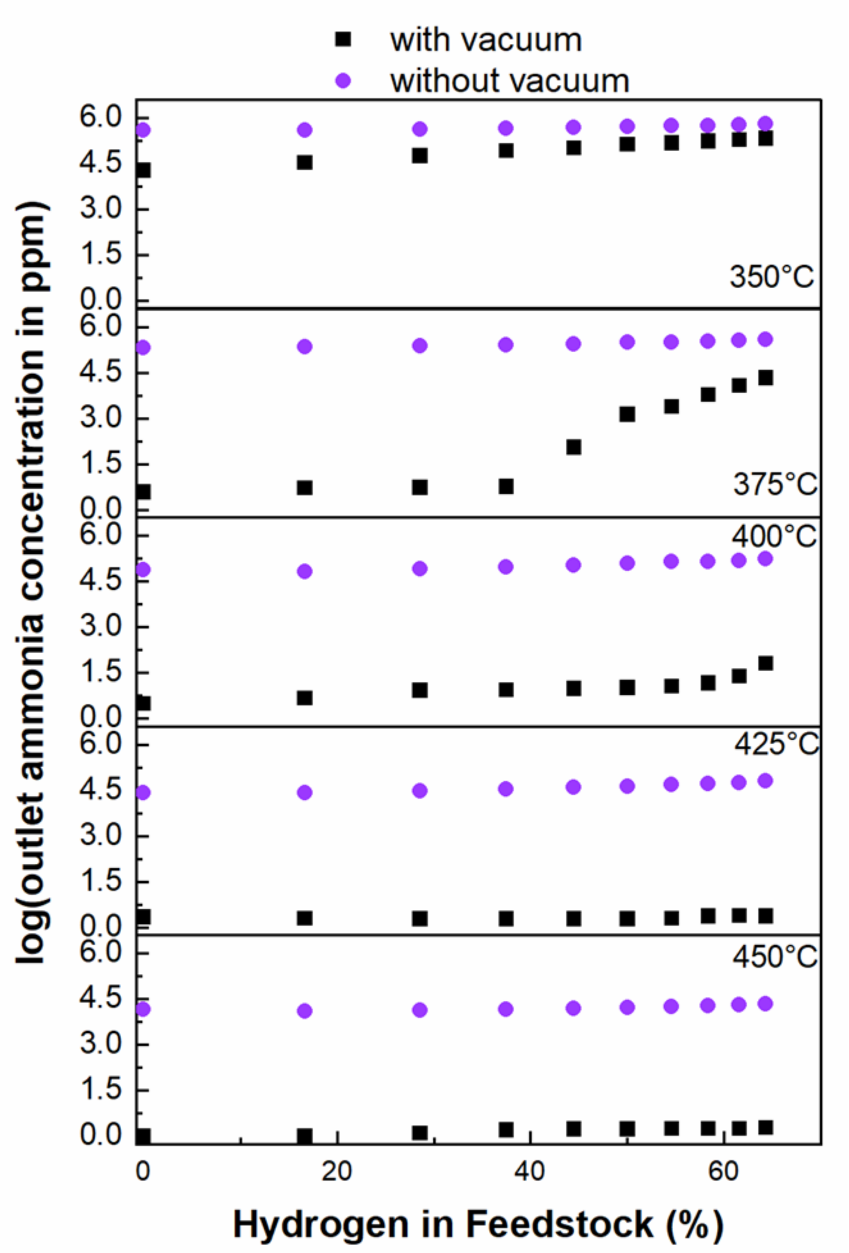

Figure S36. The effect of hydrogen in the feedstock on the cracking of ammonia in the PMR.

The conversion of the PMR system was tracked for the five temperatures tested with varied amounts of ammonia in the feedstock, with argon being used as the balance (as shown in Figure S37).

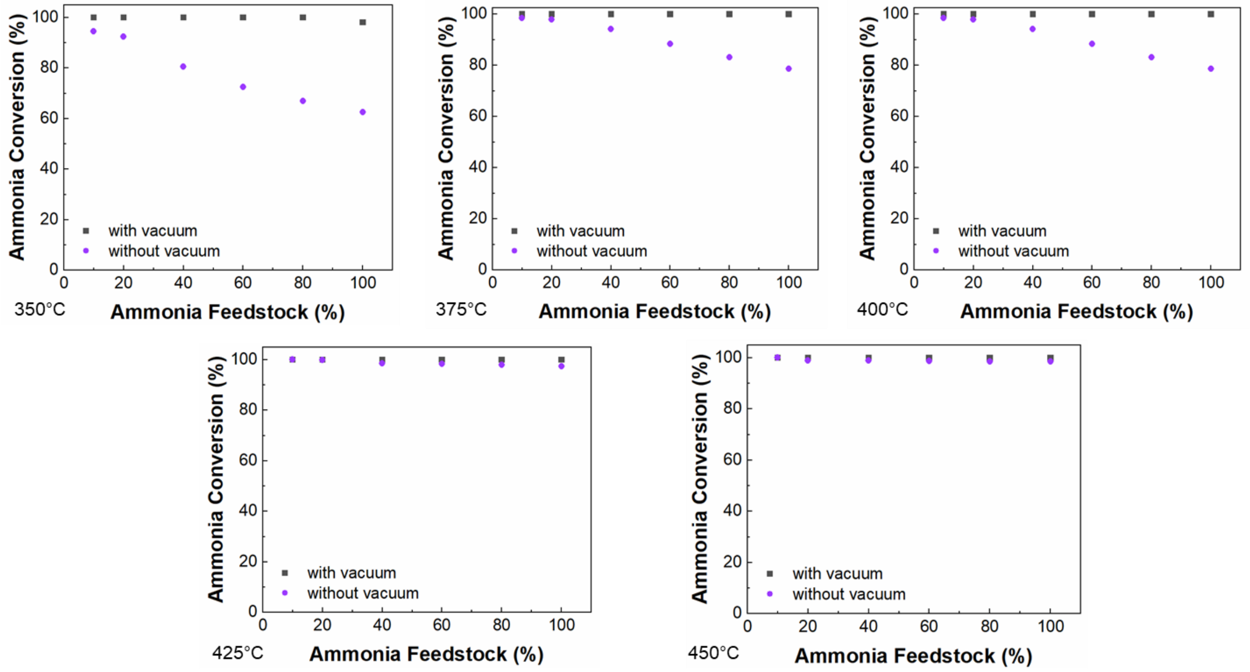

Figure S37. Conversion of ammonia at varying inlet ammonia concentrations.

The fixed bed reactor was tested for its conversion at varying pressures and is shown in Figure S38.

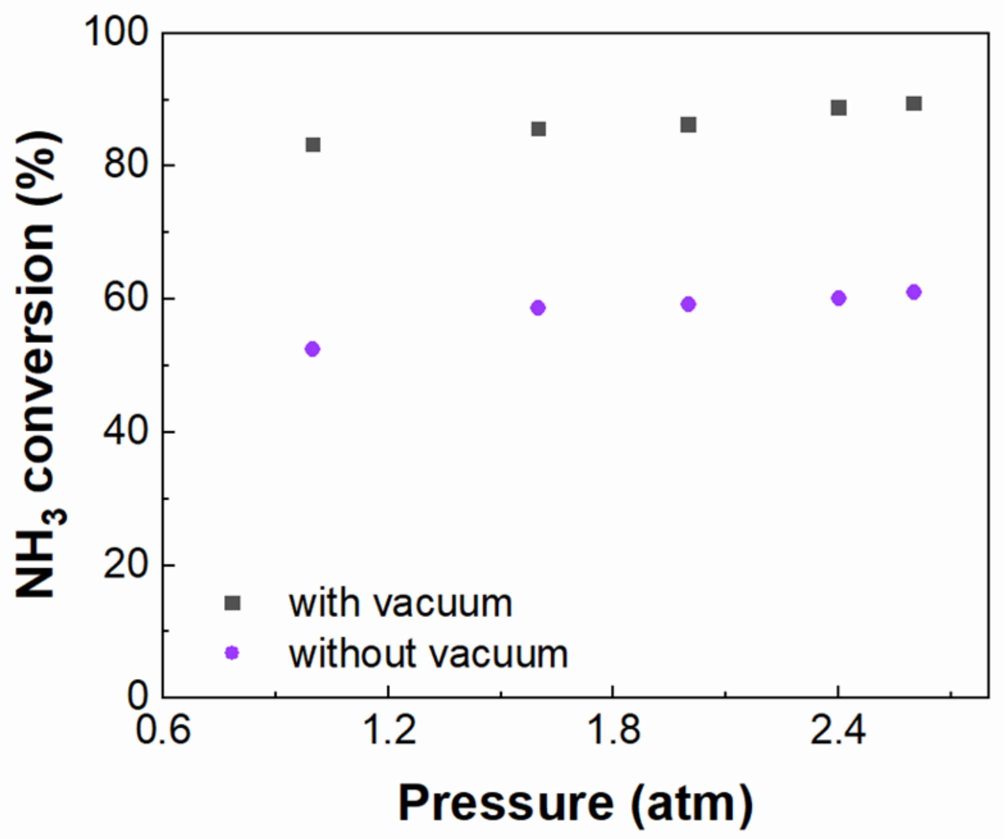

Figure S38. The effect of pressure on the conversion in a fixed bed reactor.

The stability of the PMR reaction system was tested and shown in Figure S39. The PMR system was shown to be stable over the 15 hours period with the conversion not decreasing over that time.

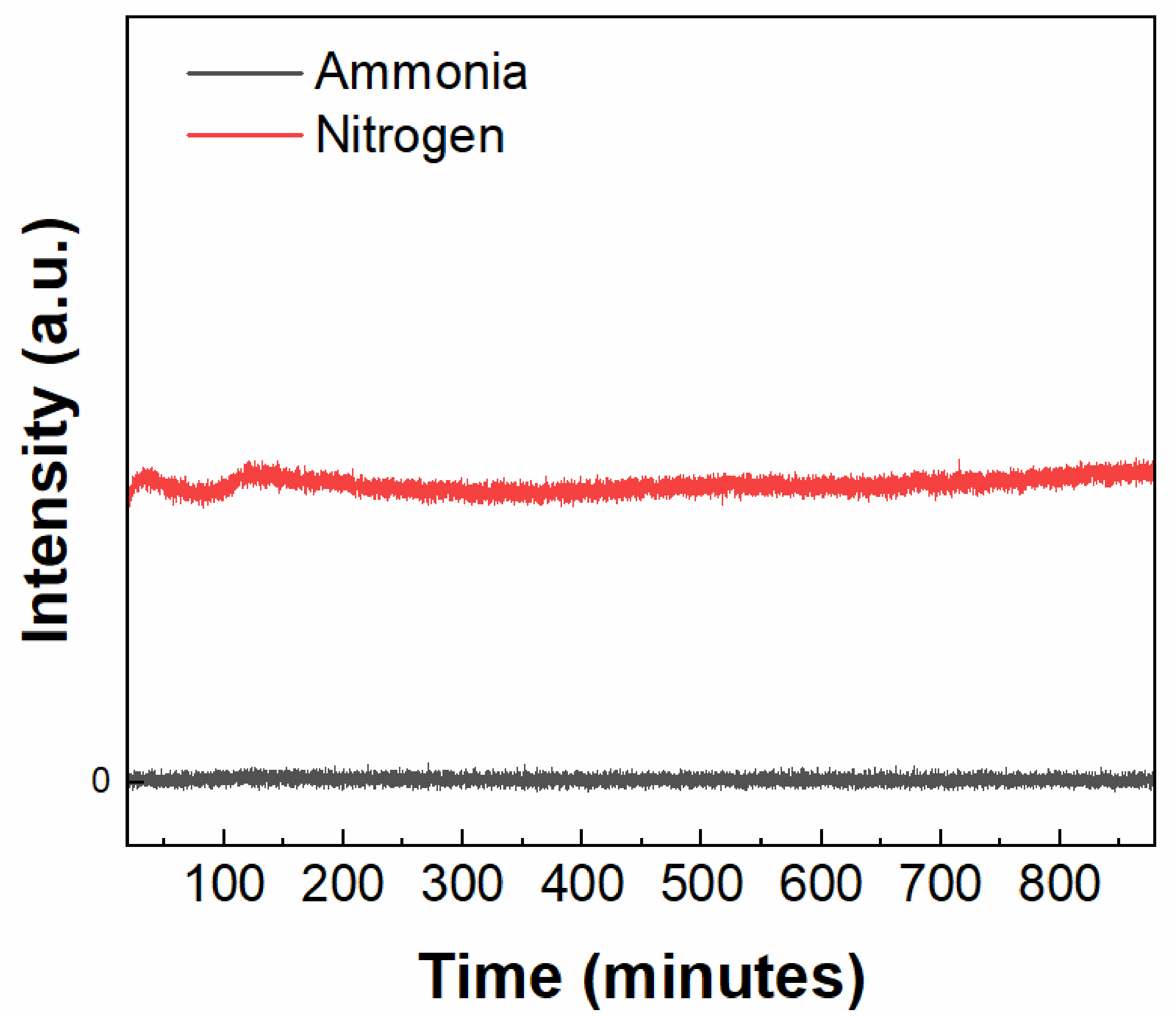

Figure S39. Stability of catalyst in permeation membrane reactor over 15 hours at 400°C.

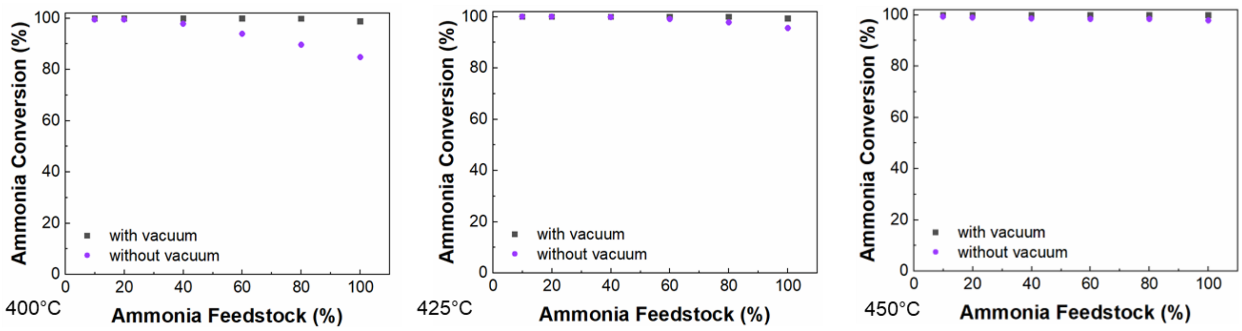

Figure S40. Ammonia-d_3 conversion at varied flow rates.

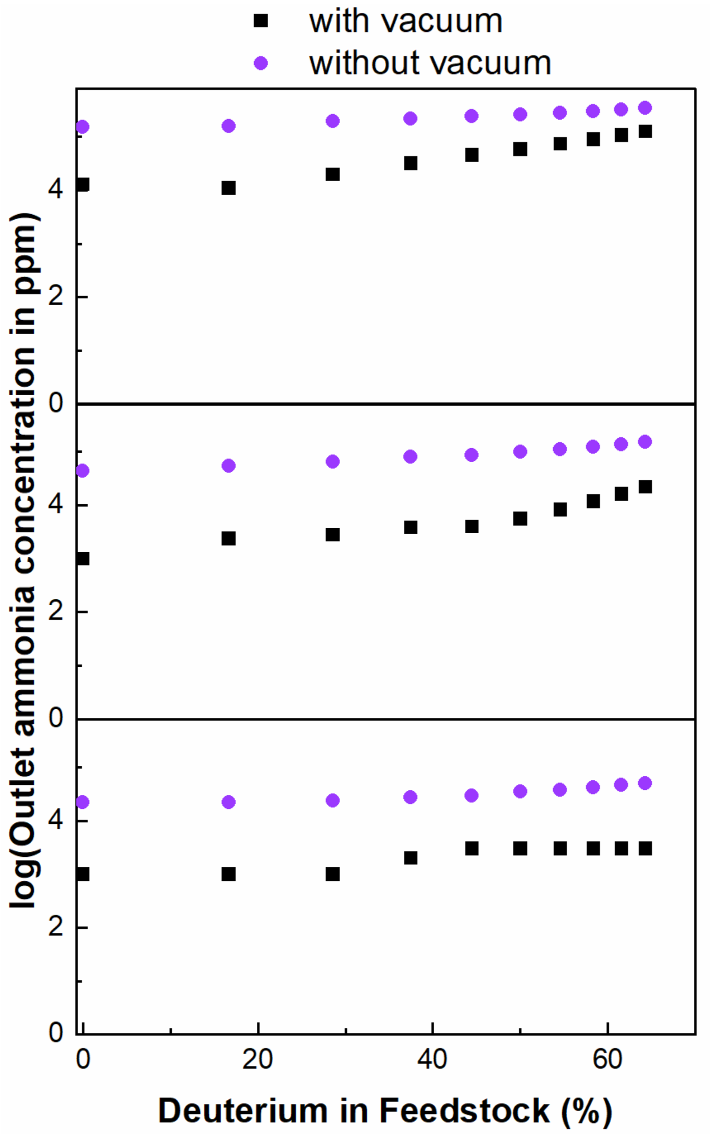

Figure S41. The effect of deuterium in the feedstock on the cracking of ammonia-d_3 in the PMR.

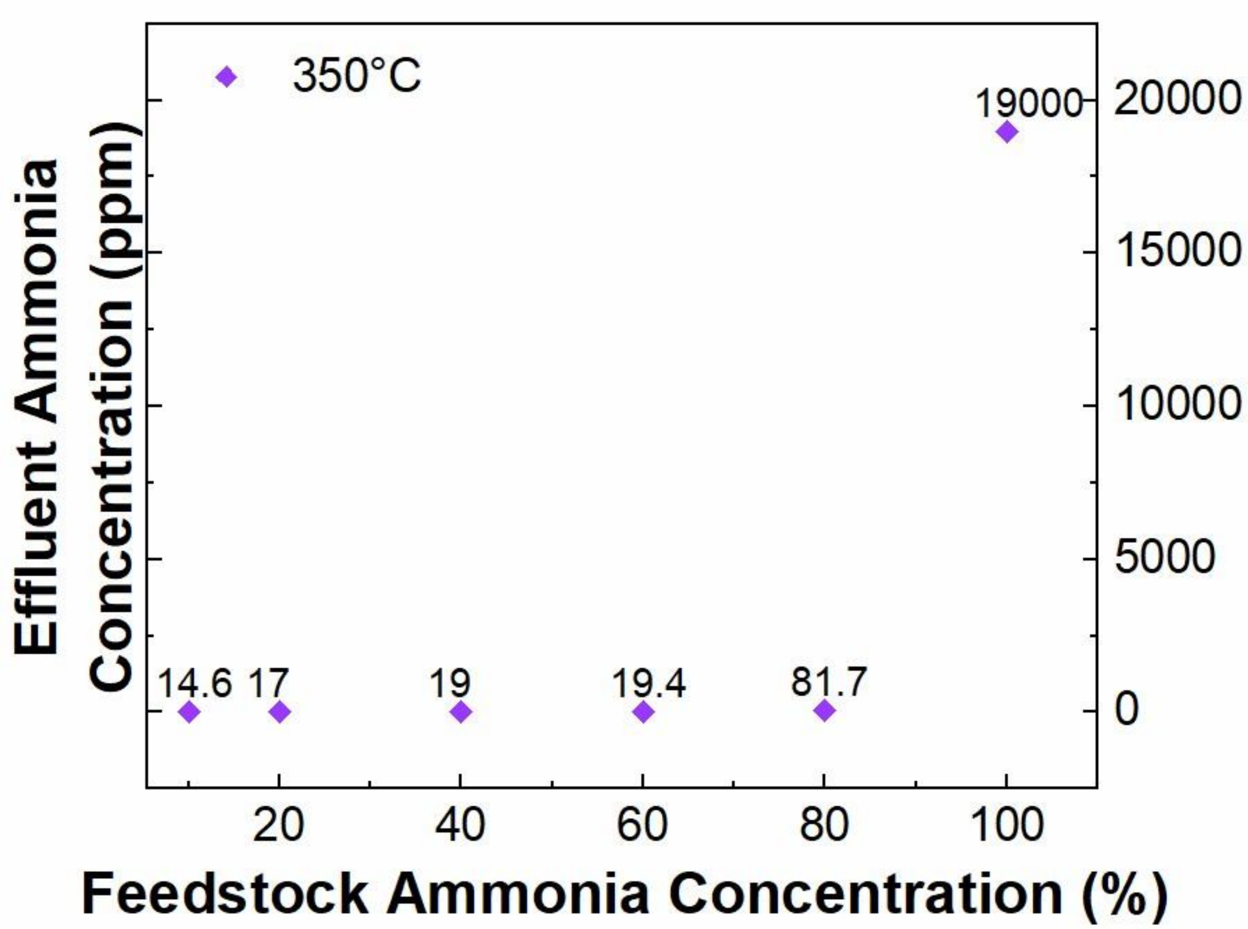

Figure S42. NH_3 cracking efficiency of a PMR at 350°C and NH_3 feedstock concentration as measured by a long pathlength tunable diode laser.

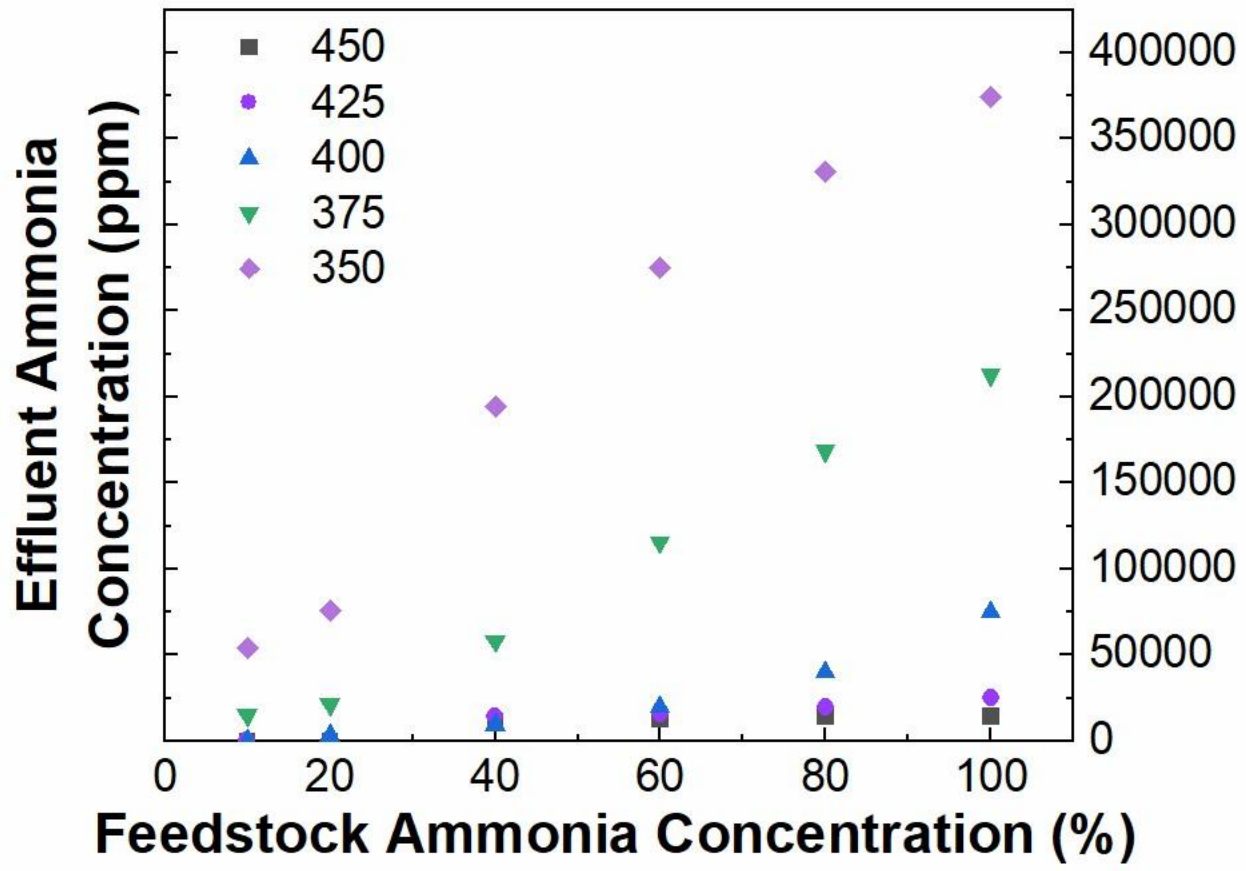

Figure S43. NH_3 cracking efficiency of a PMR as a function of temperature and NH_3 feedstock concentration as measured by a long pathlength tunable diode laser with no vacuum being pulled.

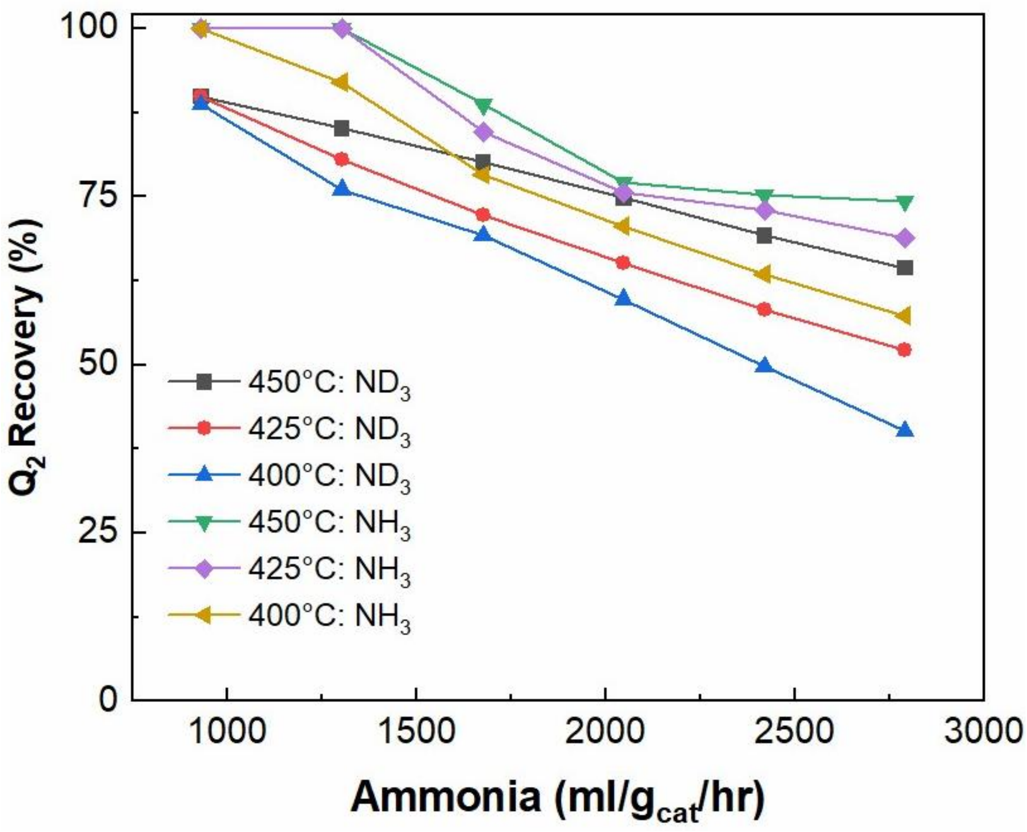

Figure S44. Q_2 recovery of the Permeation Membrane Reactor with the same conditions as in Figure 6B.
